# Paracetamol use during pregnancy — a call for precautionary action

**DOI:** 10.1038/s41574-021-00553-7

**Published:** 2021-09-23

**Authors:** Ann Z. Bauer, Shanna H. Swan, David Kriebel, Zeyan Liew, Hugh S. Taylor, Carl-Gustaf Bornehag, Anderson M. Andrade, Jørn Olsen, Rigmor H. Jensen, Rod T. Mitchell, Niels E. Skakkebaek, Bernard Jégou, David M. Kristensen

**Affiliations:** 1 Department of Public Health, University of Massachusetts School of Health Sciences, Lowell, MA USA; 2 Department of Environmental Medicine and Public Health, Icahn School of Medicine at Mount Sinai, New York City, NY USA; 3 Yale Center for Perinatal, Paediatric, and Environmental Epidemiology, Yale School of Public Health, New Haven, CT USA; 4 Department of Obstetrics, Gynecology and Reproductive Sciences, Yale School of Medicine, Yale-New Haven Hospital, New Haven, CT USA; 5 Department of Health Sciences, Karlstad University, Karlstad, Sweden; 6 Departamento de Fisiologia, Setor de Ciências Biológicas, Universidade Federal do Paraná (UFPR), Curitiba, Brazil; 7 Department of Public Health, Aarhus University, Aarhus, Denmark; 8 Department of Neurology, Danish Headache Center, Rigshospitalet-Glostrup, University of Copenhagen, Copenhagen, Denmark; 9 MRC Centre for Reproductive Health, Queens Medical Research Institute, Edinburgh, Scotland; 10 Department of Growth & Reproduction and EDMaRC, Rigshospitalet, University of Copenhagen, Copenhagen, Denmark; 11 Univ Rennes, Inserm, EHESP, Irset (Institut de recherche en santé, environnement et travail) UMR_S, 1085 Rennes, France; 12 Department of Biology, University of Copenhagen, Copenhagen, Denmark

**Keywords:** Gonadal disorders, Endocrine system and metabolic diseases, Translational research, Neurological disorders

## Abstract

Paracetamol (*N*-acetyl-*p*-aminophenol (APAP), otherwise known as acetaminophen) is the active ingredient in more than 600 medications used to relieve mild to moderate pain and reduce fever. APAP is widely used by pregnant women as governmental agencies, including the FDA and EMA, have long considered APAP appropriate for use during pregnancy when used as directed. However, increasing experimental and epidemiological research suggests that prenatal exposure to APAP might alter fetal development, which could increase the risks of some neurodevelopmental, reproductive and urogenital disorders. Here we summarize this evidence and call for precautionary action through a focused research effort and by increasing awareness among health professionals and pregnant women. APAP is an important medication and alternatives for treatment of high fever and severe pain are limited. We recommend that pregnant women should be cautioned at the beginning of pregnancy to: forego APAP unless its use is medically indicated; consult with a physician or pharmacist if they are uncertain whether use is indicated and before using on a long-term basis; and minimize exposure by using the lowest effective dose for the shortest possible time. We suggest specific actions to implement these recommendations. This Consensus Statement reflects our concerns and is currently supported by 91 scientists, clinicians and public health professionals from across the globe.

## Abstract (web summary)

A growing body of research suggests that prenatal exposure to paracetamol (APAP) might alter development and increase the risk of some reproductive, urogenital and neurodevelopmental disorders. This Consensus Statement calls for precautionary action, including a focused research effort, increasing awareness among health professionals and pregnant women and, whenever possible, minimizing use.

## Introduction

A growing body of experimental and epidemiological research suggests that prenatal exposure to paracetamol (*N*-acetyl-*p*-aminophenol (APAP), otherwise known as acetaminophen) might alter fetal development, which could in turn increase the risks of certain neurodevelopmental, reproductive and urogenital disorders^1–3^. APAP is the active ingredient in more than 600 prescription and non-prescription medications used to relieve mild to moderate pain and reduce fever (Consumer Health Products Association — acetaminophen). Sales of APAP-containing products, which are not restricted to pharmacies in many countries, are increasing worldwide^4^. Adverse effects of APAP are well known; for example, intentional overdose, unintentional overdose^5,6^ and chronic use have resulted in APAP being the leading cause of acute liver failure in children^7^ and adults^5,8^ in the Western world. Of note, APAP is widely used by pregnant women, as the FDA and EMA have long considered APAP-containing products to be of minimal risk when used as directed during pregnancy^9,10^.

As scientists, medical experts and public health professionals, we are concerned about increasing rates of neurological, urogenital and reproductive disorders. We are witnessing disturbing increases in the number of children with cognitive, learning and/or behavioural problems. For example, the US National Health Interview Survey reported that between 2009 and 2017, approximately one in six children aged 3–17 years had a developmental disability diagnosis. A 9.5% increase was observed in the overall rate of developmental disabilities between 2009–2011 and 2015–2017^11^.

Furthermore, in Western regions the prevalence of male reproductive and urogenital disorders has increased. These disorders include cryptorchidism, hypospadias and testicular germ cell cancer, together with early puberty, decreased sperm counts, levels of sex hormones and decreased fertility^12,13^. Data support the contribution of environmental exposure during fetal life, including exposure to pharmaceuticals, to these increases in rates of neurological, urogenital and reproductive disorders^13,14^.

**Fig. 1:**
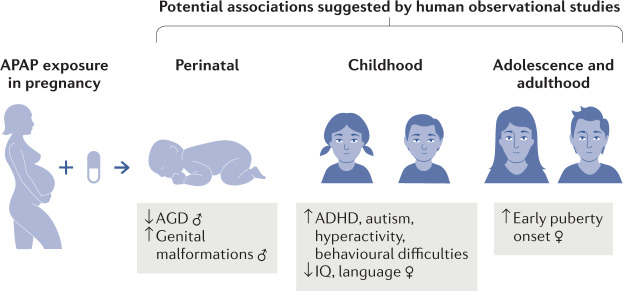
Associations between prenatal APAP exposure, reproductive and neurobehavioural development suggested from observational human studies. Human observational studies suggest prenatal *N*-acetyl-*p*-aminophenol (APAP) exposure might be associated with both reproductive and neurobehavioural abnormalities in both sexes. APAP exposure during pregnancy might increase risk of male urogenital and reproductive tract abnormalities, as studies have found an elevated risk of undescended testicles (cryptorchidism) and reduced distance between the anus and the base of the penis, a measure known as the anogenital distance (AGD). Both reduced AGD and cryptorchidism are indicators of disturbed masculinization and risk factors for reproductive disorders in later life. Prenatal APAP exposure has also been associated with earlier female pubertal development. Additionally, epidemiological studies consistently suggest prenatal APAP exposure might increase the risk of adverse neurodevelopmental and behavioural outcomes, such as attention deficit hyperactivity disorder (ADHD), autism spectrum disorder, language delay (in girls) and decreased intelligence quotient. Collectively, the studies suggest that the timing and duration of maternal APAP use are critical factors.

**Fig. 2:**
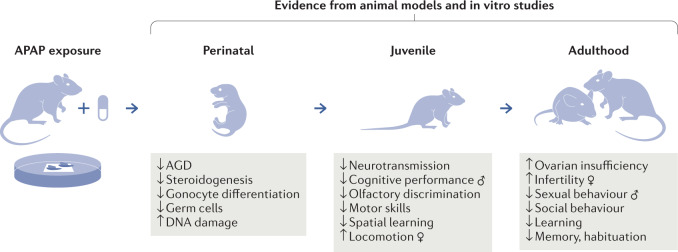
Evidence of APAP disruption of reproductive and neurological development from animal studies. In vivo, in vitro and ex vivo studies have shown that *N*-acetyl-*p*-aminophenol (APAP) directly perturbs hormone-dependent processes, which leads to disrupted reproductive development and neurodevelopment in both sexes. Fetal exposure in rodents has been shown experimentally to cause reproductive disorders of the male urogenital tract, including abnormalities in testicular function, sperm abnormalities and sexual behaviour. Experiments have shown disruption of female ovarian development resulting in reduced oocyte number and subsequent early ovarian insufficiency and subsequent reduced fertility. Fetal APAP exposure has been demonstrated to induce changes in neurotransmission in the brain manifesting in altered cognitive function, behaviour and locomotion. The studies have shown that the effect of APAP is dependent on the timing of exposure in relation to specific developmental processes and duration as well as dose. AGD, anogenital distance.

In this Consensus Statement, we summarize the epidemiological research and animal studies that have examined neurological, urogenital and reproductive outcomes that have been associated with maternal and perinatal use of APAP (Figs 1,2). Based on this research, we believe we know enough to be concerned about the potential developmental risks associated with prenatal APAP exposure and therefore call for precautionary action. We provide recommendations for a specific plan of action. We note that in this article, the terms women and men refer to cisgender women and cisgender men. The published research cited in this article does not consider pregnancy that occurs in transgender men, non-binary people or intersex people.

## Methods

This Consensus Statement is the work of an international group of experts, which included clinicians (specializing in neurology, obstetrics and gynaecology, and paediatrics), epidemiologists and basic scientists (specializing in toxicology, endocrinology, reproductive medicine and neurodevelopment). The statement was developed independently of specific societies and colleges by the 13 authors. The process of creating a Consensus Statement was initiated in parallel in Europe (D.M.K., A.M.A., C.-G.B. and B.J.) and the US (Z.L., S.H.S. and A.Z.B.). S.H.S. chaired an initial joint meeting between Z.L., A.Z.B., D.M.K., and after additional consultation with B.J., A.M.A. and C.-G.B., goals and strategy were developed for a joint statement. The expert panel decided not to include respiratory effects, as systematic reviews suggest confounding factors; for example, the presence of respiratory tract infections complicates the evidence^15–17^. D.M.K. and A.Z.B. conducted a comprehensive review of both the experimental and epidemiological literature in English available on PubMed published between 1 January 1995 and 25 October 2020, including systematic reviews, using the following search terms: ‘acetaminophen’ or ‘paracetamol’, ‘endocrine’, ‘reproduction’, ‘urogenital’, ‘neurodevelopment’, ‘attention deficit disorder’, ‘autism spectrum disorder’, ‘hypospadias’, ‘anogenital distance’ and ‘cryptorchidism’. The reference lists of identified papers were then searched for additional relevant articles. Only studies that investigated APAP as an independent exposure were included. All relevant studies are summarized in Supplementary Tables 1–5. After discussion and deliberation amongst the authors, an executive writing group (A.Z.B., S.H.S., B.J. and D.M.K.) wrote the first draft, which was circulated to all authors for critical review. Specific recommendations were accepted after extensive discussion and multiple drafts and revisions by the author group. No one outside the author group was involved in this process. Subsequently, the near final draft was circulated among clinicians, scientists and public health professionals known to the authors in relevant disciplines leading to further comments and revisions. The final statement, which takes into consideration differing international perspectives, prescribing practices and clinical considerations, was supported by 78 signees in addition to the 13 authors (Supplementary Box 1).

## APAP use during pregnancy is widespread

APAP is one of the most commonly used medications globally^2^. In the USA, APAP is estimated to be used by up to 65% of pregnant women^18,19^. Worldwide, more than 50% of pregnant women are estimated to use APAP^18,20,21^. APAP has long been considered an option by regulatory bodies such as the FDA and EMA for use in pregnancy for pain and fever when used as directed^9,10^, as NSAIDs are contraindicated for use in pregnant women in later pregnancy^22,23^. Pharmacotherapy during pregnancy involves a benefit–risk assessment, in which there is a trade‐off between the potential benefits to the mother and fetus and possible risks to the fetus^24^. The FDA has formerly given APAP a ‘B’ rating for use in pregnancy in all three trimesters, meaning that animal studies have failed to demonstrate any risks of congenital birth defects from fetal exposure and that no adequate and well-controlled studies have been performed in pregnant women^20^. In addition, the EMA has found epidemiological data inconclusive and that experimental data do not meet their standards^10,25^.

During pregnancy, the use of APAP is important for the treatment of high fever and severe pain that, left untreated, could potentially affect the developing fetus or the mother^26–31^. Fever is a well-accepted risk factor for multiple disorders, including neural tube defects and later life cardiovascular disorders^32^. Most studies, but not all^33,34^, have suggested that antipyretics such as APAP can reduce this risk^32^. In a 2020 analysis of daily APAP use in pregnant women, of women classified as APAP users, 8% reported fever as the reason for use^35^. By contrast, a review of nine cohort studies showed that fever mitigation accounted for APAP use in approximately one-third of women using APAP during pregnancy^3^. Elective APAP use for the treatment of headache, muscle pain, back pain and infection accounted for APAP use in the majority of women using APAP during pregnancy^3,35^. Importantly, studies have suggested that in the majority of women using APAP during pregnancy, its use might be without strong indications or have only limited efficacy for such conditions as chronic pain, back and knee pain, as well as headache^36–42^. In people who experience migraine headache, including women of reproductive age, studies show that migraine days can be decreased by reducing use of analgesics, including APAP^43,44^.

Research suggests that women are reluctant to use medications during pregnancy^23,45^. However, eight out of ten women take at least one prescription or over-the-counter medicine during pregnancy^45,46^ with APAP being the most common exposure (65%)^3,18^. Among pregnant women, APAP and antibiotics are believed to have the lowest risk and greatest benefit^23^. Moreover, APAP usage might be under-reported, as one study showed that when asked about pharmaceutical use, many pregnant women did not report APAP unless specifically asked^24^.

## We call for precautionary action

In this Consensus Statement, we summarize research showing adverse neurological, urogenital and reproductive outcomes associated with maternal and perinatal use of APAP. Based on this experimental and epidemiological literature (Supplementary Tables 1–4), we believe the potential for harm from continued inaction exceeds the harm that might arise from precautionary action. We recognize the limitations of the existing epidemiological literature and the need for rigorous meta-analyses (Supplementary Table 5) and therefore we call for a focused research effort. This effort should include the initiation of epidemiological and experimental studies to understand the hormonal, epigenetic and metabolic mechanisms by which APAP acts and might adversely affect development. Epidemiological studies should be designed specifically with the following goals in mind:

- Human epidemiological studies on APAP use in pregnancy should be designed to reduce confounding by indication for use.
- Human epidemiological studies on APAP use in pregnancy should be designed to control for genetic factors.
- Human epidemiological studies on APAP use in pregnancy should be designed to accurately capture exposure and outcome.
- Human epidemiological studies on APAP should be designed to examine timing, dosage and duration of exposure both prenatally and postnatally.

As this combined effort and subsequent systematic reviews will require considerable time and resources, we are proposing precautionary action that can be implemented now.

We recommend that women be counselled prior to or early in pregnancy with the following guidance:

- Pregnant women should forego APAP use unless medically indicated.
- Pregnant women should consult with their physician or pharmacist if they are uncertain whether use is indicated and before using on a long-term basis.
- Pregnant women should minimize risk by using the lowest effective APAP dose for the shortest possible time.

We recommend the following specific actions to implement the goals of raising awareness amongst patients and health-care providers:

- The 2015 FDA Drug Safety Communication recommendations should be updated based on evaluation of all available scientific evidence^9^, including both epidemiological and experimental evidence.
- The EMA Pharmacovigilance Risk Assessment Committee should review the most recent research and issue an updated Drug Safety Communication^10^, including both epidemiological and experimental evidence.
- Obstetric and gynaecological associations should review all available research and update their guidance.
- The Acetaminophen Awareness Coalition (“Know Your Dose” Campaign) should be expanded to include standardized warnings including that pregnant women should forego APAP use unless medically indicated.
- All sales of APAP-containing medications, regardless of country, should be accompanied by recommendations for use in pregnancy. This information should include warning labels on packaging of all APAP-containing medications. If possible, APAP should be sold only from pharmacies (as is currently done in France, Spain, Sweden, Finland and Iceland).

Although these recommendations might not substantially differ from advice currently given to pregnant women, we believe that APAP-specific risk communication between health professionals and pregnant women is warranted due to the high prevalence of use and a widespread perception of negligible risk. APAP use should be minimized but might in some situations, such as high fever and/or severe pain, be the course of action with the lowest risk.

## APAP is an endocrine disruptor

Chemicals that disrupt the endocrine system are concerning because they can interfere with the activity of endogenous hormones that are essential for healthy neurological, urogenital and reproductive development^2,47,48^. APAP is known to readily cross the placenta and blood–brain barrier^49,50^. During pregnancy, changes occur in APAP metabolism, which might make pregnant women and their fetus more vulnerable to toxic effects. For instance, the molar dose fraction of APAP that is converted to the oxidative metabolite *N*-acetyl-*p*-benzoquinone imine might be increased during pregnancy^51,52^.

The analgesic and antipyretic properties of APAP are still not fully understood. However, several lines of evidence suggest that APAP acts both in the periphery and centrally through several mechanisms. For example, one of the ways APAP is believed to relieve pain is through inhibition of prostaglandin signalling^53^. Furthermore, APAP inhibits serotonergic mechanisms in clinical studies^54^. APAP also acts as a prodrug for analgesic metabolites^55^; in experimental studies, these metabolites activate serotonergic, opioidergic, vanilloid and cannabinoid receptors, as well as transient receptor potential channels^53,56^. Prostaglandins are lipid compounds with physiologically important roles in the development of the gonads in both sexes and the development of the brain^57–59^; therefore, some of the disrupting effects of APAP are probably mediated through this pathway. Moreover, increasing clinical evidence suggests that the action of APAP in inhibiting prostaglandin signalling in the third trimester can lead to ductus arteriosus constriction, a condition that might result in fetal loss or life-threatening cardiac failure in the newborn^60^.

In vivo, in vitro and ex vivo studies have shown that APAP directly perturbs hormone-dependent processes, including inhibition of androgen production and increased oestrogen production, disruption of steroidogenesis, depletion of sulfated sex hormones, perturbation of immune function, induction of oxidative stress and indirect activation of the endocannabinoid system^1,2,61–65^. Independently of APAP, these processes have been implicated as mechanisms related to the development of neurodevelopmental^66–76^ and reproductive disorders^2^. In addition to potential effects on neuronal and reproductive development, a combination of clinical studies together with experimental work in animal models and cell lines has also suggested that APAP exposure during pregnancy might decrease fetal haematopoietic stem cell numbers alter steroidogenesis in the placenta and induce placental damage^65,77–80^.

Studies of intrauterine APAP exposure have found effects on development in both mouse and rat models. In these studies, doses ranged from the maximum safe dose for a pregnant woman of 50 mg/kg per day to 1,430 mg/kg per day (Supplementary Tables 2, 4)^81,82^. The majority of studies have used between 200 and 400 mg/kg per day, which is well within the appropriate range for comparing animal and human exposure studies, if the difference in body size is taken into consideration. Hence, for such studies a system of allometry (how anatomy, physiology and behaviour change based on body size) based on body surface area is normally applied, where mouse and rat dose data are divided by a factor of 12.33 and 6.2, respectively, to reach the equivalent human dose^83^.

Taken together, APAP has many of the key characteristics for hazard identification of an endocrine-disrupting chemical^84^. However, understanding the exact mechanisms that lead to a particular outcome in humans is complicated as APAP potentially interacts with several critical pathways during development, such as prostaglandins and steroids. Furthermore, the mechanisms leading to APAP-induced reproductive outcomes are probably not the same as those leading to neurological outcomes. In addition, the effect of APAP is dependent on the timing of exposure; for example, any effects on testicular masculinization might not necessarily overlap with the effects on brain masculinization.

## Potential urogenital and reproductive effects

Concerns about the safety of APAP in relation to urogenital and reproductive effects have not been addressed in reviews by governmental authorities such as the FDA, or obstetric and gynaecological associations^9,85,86^. Although some studies have shown no APAP-induced effects, an increasing body of evidence suggests that APAP has the ability to disrupt animal and human reproductive tract development, from fetal life to adulthood in both sexes^1,2^. Fetal exposure in animal models has been shown experimentally to cause disorders of the male urogenital tract through reduction of androgen action^2^. Furthermore, experimental models have consistently shown disruption of ovarian development, which results in reduced fertility at the same dose or close to the dose used by pregnant women^87,88^.

### The epidemiological evidence of urogenital and reproductive effects

The relationship between prenatal APAP exposure and urogenital and reproductive abnormalities has been investigated in 11 observational studies in six cohorts including over 130,000 mother–child pairs from different parts of the world^24,89–98^ (for a comprehensive review of the literature including effect sizes see Supplementary Table 1). Findings from five of these studies suggest that prenatal APAP exposure is associated with male urogenital and reproductive tract abnormalities, by showing increased risk of male undescended testicles (cryptorchidism)^24,89,90^ and a reduced anogenital distance (AGD)^91,92^. An additional study has suggested an association between prenatal APAP exposure and early female puberty^93^. Four studies have found no increased risk of hypospadias from prenatal APAP exposure^94–97^.

AGD is the distance between the anus and the base of the penis, which is an indicator of the degree of masculinization of the genitals^51,91,92,99^. Both AGD and cryptorchidism are risk factors for reproductive disorders in later life^13^. Reduced AGD has been found in boys following exposure to APAP during weeks 8–14 of gestation, which coincides with the timing of the masculinization programming window. This period is when male reproductive development (including AGD) is considered to be programmed^91,100^. Importantly, reduced AGD is recapitulated in mouse and rat experimental studies when animals are exposed in the equivalent rodent programming window (explained later). Another study has demonstrated reduced AGD in boys exposed to the combination of APAP and NSAIDs during pregnancy, suggesting a potential additive effect as exposure to APAP alone did not result in a significant difference in AGD^92^. Similar additivity with other analgesics has been seen for cryptorchidism, where the association is strongest among mothers using more than one analgesic during pregnancy^24^. Moreover, exposure to APAP for >2 weeks increased the risk of cryptorchidism^24^. Most associations for cryptorchidism are seen following long-term APAP exposure (>2 weeks) during late first to early second trimester, which is consistent with the critical time windows for development^2,91^. Thus, these data suggest that the timing and duration of maternal APAP use are critical factors and that short-term APAP use might be of limited risk.

Although not directly related to evidence of the effects of APAP in utero, studies have also suggested that adult men who used APAP had increased time to pregnancy, decreased testosterone production and had sperm abnormalities, including DNA fragmentation^101,102^. Moreover, exposure of ex vivo adult testes to APAP has similarly shown a negative effect on testosterone production^103^. A single cohort study that investigated prenatal APAP exposure and female reproductive outcomes found increasingly earlier attainment of markers of female pubertal development (for example, pubic and axillary hair) with increasing number of weeks of prenatal APAP exposure, in a dose-responsive manner^93^.

These observational studies controlled for numerous confounding factors but were potentially limited by residual confounding and exposure and outcome misclassification. Confounding by indication for use was controlled for in nine studies^24,89,90,93–95,97,101,102^. Exposure assessment relied on maternal self-reported APAP use in all 11 studies. In these cohorts exposure information was collected in a timely manner to attempt to minimize exposure misclassification and recall bias. Importantly, such misclassification is most likely to be non-differential and result in underestimation of the true effect, rather than to represent spurious associations^104^.

Together, increasing evidence suggests that prenatal APAP exposure is associated with male urogenital and reproductive tract abnormalities (Fig. 1). Inconsistencies between studies are probably due to differences in assessment methodologies of exposure and outcomes. Moreover, the fact that APAP is a less potent anti-androgen than other pharmaceuticals (for example, ketoconazole) probably means that only large studies would be sufficiently powered to detect effects.

### The experimental evidence of urogenital and reproductive effects

Consistent with evidence from epidemiological studies, exposure to APAP has been linked to abnormalities in testicular function, sperm abnormalities and the development of male reproductive disorders across a range of studies involving in vitro, ex vivo and in vivo models (Supplementary Table 2). These data suggest that several mechanisms of action result in decreases in hormones critical for normal reproductive development and inhibition of germ cell proliferation and differentiation. For example, experimental studies have suggested that APAP can reduce testosterone production in the human fetal testis^105^. Treatment of pregnant mice and rats with APAP has been found to cause urogenital abnormalities, such as reduced AGD in male offspring that is coupled to decreased hormonal levels during the masculinization programming window^24,64,105^. Differences and inconsistencies remain between studies that might be related to several factors, including species, strain, age, developmental stage, dose, duration, and route and schedule of administration, among others. However, strong evidence from rodent studies and experiments with human cells and tissue performed in several independent laboratories shows that both acute (for example, 24 h) and long-term (for example, 1 week) exposure to APAP results in a reduction in fetal androgens^24,105^.

Both acute exposure (24 h) and long-term exposure (for example, 1 week ex vivo or from 7 days *post coitum* to birth in vivo) of rodent and human fetal gonadal tissue to APAP have been demonstrated to adversely affect germ cell development and proliferation^81,106^. Additionally, prenatal exposure of mice to APAP from 7 days *post coitum* to birth seems to impair male sexual behaviour in adulthood by disrupting sexual neurobehavioural programming^107^.

Four independent research teams have found consistently that prenatal APAP exposure can reduce female reproductive health and fertility^81,87,88,106,108,109^. These teams utilized different models in both rats and mice with APAP exposure at doses equivalent or close to the maximal human recommended dose from 7 days *post coitum* to birth or from 13.5 days *post coitum* to birth. The combined data show that APAP exposure results in the reduction of primordial germ cells and delayed meiotic entry, which leads to a decreased number of follicles in adult ovaries and subsequent infertility through early-onset ovarian insufficiency^81,106,108,109^. Importantly, the effects of APAP on female development have not yet been properly investigated in human observational studies.

## Potential neurodevelopmental effects

The developing human brain is uniquely vulnerable to exposure to toxic chemicals. Critical windows of developmental vulnerability occur in utero, and during infancy and early childhood^110^. During these sensitive life stages, certain chemicals can cause permanent brain injury at low exposure levels^111^.

### The epidemiological evidence of neurodevelopmental effects

The relationships between prenatal APAP exposure and adverse neurodevelopmental outcomes have been investigated in 29 observational studies in 14 cohorts including over 220,000 mother–child pairs from different parts of the world^19,112–138^ (for review of the literature including effect sizes see Supplementary Table 3). Of these studies, 26 identified positive associations with APAP exposure during pregnancy and a range of clinically assessed and parent-reported neurodevelopmental outcomes, primarily attention deficit hyperactivity disorder (ADHD)^115,119–121,123,126,129–134,137,138^ and related behavioural abnormalities^19,116–118,135^, but also autism spectrum disorder (ASD)^121,124,130^, language delays^117,118,127,136^, decreased IQ^113^, cerebral palsy^122^, oppositional–defiant disorder^125^, decreased executive function^112,114^ and conduct disorders^125^. Effect sizes were generally modest but because exposure is widespread^1,107^, even a small effect size could translate into a large number of affected children. Dose–response has been investigated in 19 studies^112–114,116–122,124,127–130,133,136–138^ and of these, 16 studies^112,114,116–122,124,127,129,130,133,136,137^ identified a dose–response association, whereby increased duration of exposure was associated with increased risk. In many of these studies, associations were weak for short-term exposure suggesting that short-term use might be of limited risk. As with reproductive and urogenital outcomes, exposure timing is important, as the highest risk seemed to occur from exposure during the second and third trimesters of pregnancy (with some exceptions^112–114^). Two studies also investigated APAP exposure during infancy. One study of infant exposure identified associations with decreased mid-childhood executive function and poor behaviour^114^, whereas the other found no association with cognitive development outcomes^19^.

These 29 observational studies were limited by potential confounding, including by indication for APAP use, by genetic factors and by bias introduced by exposure and outcome misclassification, as well as study participant loss to follow-up^139^. Several analytical techniques have been used to control for confounding by indication, with results largely remaining unchanged^3,41^. Similar disease risk observed across different indications supports a causal association, as different indications (for example, fever and back pain) are unlikely mechanistically to affect disease risk in similar ways^140^. Using methods such as sibling control design, polygenic risk scores and negative controls, efforts were made to control genetic confounding in 16 studies with little effect on the reported associations in all but two of these studies^115,116^. APAP exposure assessment relied on maternal self-report in 24 studies, on biomarkers in five studies^127–130,137^ and on prescription records in one study^138^. Although one study that used both biomarkers and self-reported exposure suggested that the two measures of exposure were correlated^127^, exposure misclassification remains a concern in studies using maternal self-report^24^. Timely collection of exposure information would probably minimize such exposure misclassification and recall bias. Importantly, misclassification, as mentioned above, is most likely to be non-differential and result in underestimation of the true effect, rather than to represent spurious associations^104^. The underestimation could be substantial, as evidenced by the far stronger associations reported in biomarker studies^127,129,130,137^ than in studies relying on self-report.

Two notable studies overcame some of the important limitations of earlier studies. A 2021 study evaluated the association between levels of APAP metabolites in umbilical cord plasma (direct evidence of fetal exposure^141^) and physician-diagnosed childhood ADHD, ASD and other developmental disabilities, using data from the Boston Birth Cohort^130^. Cord plasma APAP metabolite concentrations in the first tertile compared with the second and third tertiles were associated with a more than twofold higher odds of an ADHD diagnosis and up to a threefold higher odds of an ASD diagnosis. Sensitivity analyses and subgroup analyses found consistent associations between APAP metabolite concentrations and ADHD and ASD across strata of potential confounders, including maternal indication, substance use, preterm birth, and child age and sex.

In a 2020 prospective cohort study conducted in Québec, Canada, children exposed to APAP prenatally (as measured in meconium) were at increased risk of physician-diagnosed ADHD and hyperactivity, which was indicated by resting-state brain connectivity at ages 6 and 7 years^137^. Compared with no APAP, detection of APAP in meconium was associated with twice the odds of ADHD. A dose–response association was also detected. Prenatal APAP exposure was also associated with increased negative connectivity between the left prefrontal cortex (frontoparietal seed) and the right precentral gyrus, which mediated the association of APAP with hyperactivity. The authors stated “these results suggest that prior studies may have been biased towards the null by inaccurate maternal recall. Thus, the association between prenatal acetaminophen and ADHD may be even stronger than previously estimated”^137^. This study established for the first time an association between prenatal APAP exposure and a physical manifestation of neurological alteration. This study not only potentially identifies an underlying mechanism, but also reduces concern that associations found in earlier studies might have been due to diagnostic inaccuracy introduced by suboptimal or subjective outcome measurement^139^. A limitation of the study is that it did not control for indication; however, when effect sizes are large, as seen in this study, residual confounding by uncontrolled factors is a less likely explanation for identified associations^142^.

Present biomarker studies are not without limitations in the assessment of exposure. For example, present standard targeted methods are based on analysing free APAP and phase II conjugates after enzymatic deconjugation^143^. This method captures only part of the metabolic pathway and leaves a fairly short window to assess APAP due to its short half-life (4–6 h)^141,143,144^, which can lead to underestimation of actual exposure. Thus, a 2021 study suggested that biomarkers identified with standard methods used in biomonitoring are inadequate for human biomonitoring of a non-persistent chemical such as APAP and result in underestimation of actual exposure^141^.

### The experimental evidence of neurodevelopmental effects

Experimental animal studies have suggested that perinatal APAP exposure, even at low therapeutic doses, increases the risk of brain and behavioural abnormalities in rodents^51,145–151^, supporting the epidemiological evidence (Supplementary Table 4). A 2019 study suggested that APAP enters the developing rat brain and cerebrospinal fluid in higher amounts than the adult brain. Long-term (5 days) fetal exposure resulted in even higher transfer rates than short-term exposure, which might lead to accumulation of APAP in the fetal brain^50^. Consistent with the epidemiological data, studies have demonstrated that the strongest effects of long-term use and exposure occur at a time equivalent to the beginning of the third trimester of pregnancy and the time around birth in humans^69,148^.

APAP effects on the cannabinoid and prostaglandin pathways alone or in combination might be the possible mechanisms^1,62,65,69,152–154^. The antipyretic effect of APAP involves inhibition of prostaglandin-synthesizing cyclooxygenase enzymes in the brain^155^. Prostaglandin E2 is an endogenous lipid molecule involved in normal brain development, regulating cerebellar development and inducing masculinization of the preoptic area of the brain^70^. Emerging clinical and molecular research has provided compelling evidence that abnormal cyclooxygenase 2 and prostaglandin E2 signalling is associated with ASD-related pathology and behaviours^68,69,154,156–158^. Other data suggest that the analgesic effect of APAP acts through the endocannabinoid system^159^. The endocannabinoid system is a complex network of lipid signalling pathways that have an important role in the developing nervous system^160^. Alterations of the endocannabinoid system have been found in both the brain and the immune system of humans with ASD^161,162^. Studies in mice have demonstrated the emergence of ASD-like behaviours following diverse genetic or pharmacological manipulations targeting the endocannabinoid system^160^.

As with reproductive studies, inconsistencies between studies might relate to factors such as species, strain, age, dose, duration of exposure, and route and schedule of administration. However, a particular obstacle is the difficulty in translating human outcomes, such as ADHD and ASD, to behaviour in an animal model. Future studies should include evaluation of brain and behavioural effects in higher order species, from both prenatal and early life exposure, for specific indications and exposure windows^153^.

## Conclusions

This APAP Consensus Statement is a call to prioritize research initiatives and to provide evidence-based medical guidance for APAP use by pregnant women, with the goal of creating awareness so women can make informed decisions that will lead to minimizing APAP exposure. We therefore call for agencies such as the FDA and EMA and appropriate obstetric and gynaecological societies to review all available data covering both epidemiological and experimental studies, so an evidence-based evaluation of the risk can be made available to inform patients and their health-care professionals. The limitations that we identified in the existing literature should be addressed in well-designed research that accurately captures medication use during pregnancy and minimizes potential confounding by indication and exposure misclassification.

We here recognize our professional and social responsibility to take this action, even in the face of uncertainty, in light of the serious consequences of inaction. This call to action is consistent with the 2016 Targeting Environmental Neuro-Developmental Risks (TENDR) Consensus Statement^47^, which we support:

“We as a society should be able to take protective action when scientific evidence indicates a chemical is of concern, and not wait for unequivocal proof that a chemical is causing harm to our children. Evidence of neurodevelopmental toxicity of any type — epidemiological or toxicological or mechanistic — by itself should constitute a signal sufficient to trigger prioritization and some level of action.”^47^.

In addition, a new opinion statement issued by the American College of Obstetricians and Gynecologists suggests that gynaecologists should screen patients for exposure to environmental chemicals before and during pregnancy and counsel on how to minimize risks. We support this statement, which is consistent with our recommendations^163^.

APAP is a modifiable exposure. We recognize that limited medical alternatives exist to treat pain and fever; however, we believe the combined weight of animal and human scientific evidence is strong enough for pregnant women to be cautioned by health professionals against its indiscriminate use, both as a single ingredient and in combination with other medications. We recommend that APAP should be used by pregnant women cautiously at the lowest effective dose for the shortest possible time. Long-term or high-dose use should be limited to indications as advised by a health professional. Packaging should include warning labels including these recommendations. Given the high prevalence of APAP use by pregnant women, the public health implications of use reduction might be substantial.

## Supplementary information

Supplementary Information
