# Supplementary material for: Paracetamol use during pregnancy — a call for precautionary action
Source: Nat Rev Endocrinol. 2021 Sep 23;17(12):757–66. doi: 10.1038/s41574-021-00553-7 (PMC8580820; doi:10.1038/s41574-021-00553-7)
Supplement: Supplementary file 1 — Supplementary Information [file 41574_2021_553_MOESM1_ESM.pdf]

nature portfolio

https://doi.org/10.1038/s41574-021-00553-7

Supplementary information

Paracetamol use during pregnancy — a call for precautionary action

In the format provided by the authors and unedited

Supplementary Box 1: Signatories

Signee	Affiliation
1. Natalie Aneck-Hahn MD	Director, Environmental Chemical Pollution and Health Research Unit, Deputy Director: Medical Natural Sciences, Department of Urology, Steve Biko Academic Hospital, School of Health Systems and Public Health, Faculty of Health Sciences, University of Pretoria, South Africa.
2. Jes Olesen MD Dr Med	Professor of Neurology, University of Copenhagen, Danish Headache Center, Department of Neurology, Righospitalet, Copenhagen University Hospital, Copenhagen, Denmark.
3. Anders Hay-Schmidt PhD	Professor, Department of Odontology, Faculty of Healthand Medical Sciences, University of Copenhagen, Denmark.
4. Karsten Kristiansen PhD	Professor of Molecular Biology, Department of Biology, University of Copenhagen, Denmark/Professor and Director, Institute of Metagenomics, BGI-Shenzhen, China.
5. Gunnar Toft PhD	Associate Professor, Department of Clinical Medicine - Department of Clinical Epidemiology, Aarhus University, Denmark.
6. Terje Svingen PhD	Head of Research Group, Associate Professor, NationalFood Institute, Research group for Molecular and Reproductive Toxicology, Technical University of Denmark, Denmark.
7. Anna-Maria Anderson PhD	Director of the National Danish Centre on Endocrine Disrupting Chemicals, Department of Growth and Reproduction, Rigshospitalet, Copenhagen University Hospital, Copenhagen, Denmark.

8. Anders Juul MD Dr Med	Head of Department, Professor of Growth and Reproduction, Department of Growth and Reproduction, Rigshospitalet, Copenhagen University Hospital, Copenhagen, Denmark.
9. Katharina Main MD PhD	Professor of Pediatrics, Department of Growth and Reproduction, Rigshospitalet, Copenhagen University Hospital, Copenhagen, Denmark.
10. Hanne Frederiksen PhD	Senior scientist, Department of Growth and Reproduction, Rigshospitalet, Copenhagen UniversityHospital, Copenhagen, Denmark.
11. Gorm Greisen MD Dr Med	Professor of Pediatrics, Department of Neonatology, Rigshospitalet, Copenhagen University Hospital, Copenhagen, Denmark.
12. Søren Ziebe PhD	Head of Department, Professor in Clinical Embryology, Department of Fertility, Rigshospitalet, Copenhagen University Hospital, Copenhagen, Denmark.
13. Morten Rønn Pedersen PhD	Head of Laboratory, Clinical Embryologist, Department of Fertility, Rigshospitalet, Copenhagen University Hospital, Copenhagen, Denmark.
14. Tina Kold Jensen MD PhD	Professor, Department of Environmental Medicine, Institute of Public Health, Department, University ofSouthern Denmark, Denmark.
15. Cecilia Ramlau-Hansen PhD	Professor, Department of Public Health, Aarhus University, Denmark.
16. Andrea Ernst PhD	Department of Public Health, Aarhus University, Denmark.
17. Casper Hagen MD PhD	Department of Growth and Reproduction, Rigshospitalet, University of Copenhagen, Denmark.

18. Margit Bistrup Fischer MD	Department of Growth & Reproduction, Rigshospitalet, University of Copenhagen, Copenhagen, Denmark.
19. Andreas Kortenkamp PhD	Professor of Human Molecular Toxicology, Institute of Environment, Health and Societies, Department of Life Sciences, Brunel University London, UK.
20. Paul Fowler PhD	Professor, Director of the Institute of Medical Sciences and Chair in Translational Medicine, University of Aberdeen, UK.
21. Karine Audouze PhD	Associate Professor of Systems Toxicology, Environment and human health, Université de Paris, France.
22. Nathalie Rives PhD	Professor, Normandie Univ, Rouen University Hospital, Biology of Reproduction-CECOS laboratory, Rouen, France.
23. Robert Barouki MD PhD	Professor of Biochemistry, French Institute of Health and Medical Research (Inserm), Unit of Toxicology, Pharmacology and Cell Signaling, University Paris Descartes, France.
24. Xavier Coumoul PhD	Professor of Biochemistry and Toxicology, University Paris Descartes, France.
25. Louis Bujan MD PhD	Professor of Reproductive Medicine, Director of the Human Fertility Research group, Head of the Femme-Mère-Couple hospital, Toulouse University Hospital, France.
26. Yves le Bouc MD PhD	Professor of Physiology, French Institute of Health and Medical Research (Inserm), Unité Mixe Recherche Scientifique 938, Centre de Recherche St-Antoine (CRSA), Sorbonne Université, Paris, France.

27. Gabriel Livera PhD	Professor, Head of the Laboratory of Development of the Gonads, University of Paris, France.
28. Aleksander Giwercman MD PhD	Professor of Reproductive Medicine, Lund University, Malmö, Sweden.
29. Yvonne Giwercman PhD	Professor of Molecular Genetic Reproductive Medicine, Department of Translational medicine, Lund University, Malmö, Sweden.
30. Jorma Toppari MD PhD	Professor of Pediatrics, Chairman of Physiology, University of Turku, Finland.
31. Ilpo Huhtaniemi MD PhD	Professor Emeritus, Reproductive Endocrinology, Institute of Reproductive and Developmental Biology, Imperial College London, UK.
32. Serge Nef PhD	Professor, Department of Genetic Medicine and Development, Switzerland Institute of Genetics and Genomics of Geneva, University of Geneva, Switzerland.
34. Josephine Bowles PhD	Associate Professor, Institute for Molecular Bioscience,School of Biomedical Sciences, The University of Queensland, Australia.
35. Francisco Paumgartten MD PhD	Professor of Toxicology and Public Health, National School of Public Health, Oswaldo Cruz Foundation (FIOCRUZ), Rio de Janeiro, Brazil.
36. Wilma de Grava Kempinas PhD	Professor of Reproductive Toxicologist and Embryology,Institute of Biosciences of Botucatu, Sao Paulo State University, Brazil.
37. Daniela Ceccatto Gerardin PhD	Department of Physiological Sciences. State University of Londrina, Londrina, Paraná, Brazil.
38. Estefania Moreira PhD	Professor of Pharmacology and Toxicology, Department ofPhysiological Sciences, State University of Londrina, Brazil.

39. Eliane Dallegrave PhD — Associate Professor, Department of Pharmacosciences,Federal University of Health Sciences of Porto Alegre, Porto Alegre, Brazil.

40. Graziela S. Ceravolo PhD — Professor of Pharmacology, Department of PhysiologicalSciences, State University of Londrina, Londrina, Brazil.

41. Martha Herbert MD PhD — Pediatric Neurologist, Department of Neurology, Massachusetts General Hospital, Boston MA, USA.

42. Stephen Schultz PhD — Department of Cellular and Integrative Physiology, School of Medicine, University of Texas Health Science Center, San Antonio, San Antonio, USA.

43. Richard E. Frye MD PhD — Associate Professor, Pediatric Neurologist, Chief of Division of Neurodevelopmental Disorders, BarrowNeurological Institute at Phoenix Children's Hospital, University of Arizona, College of Medicine, USA.

44. Cynthia Nevison PhD — Institute for Alpine and Arctic Research, University of Colorado, USA.

45. Michael Belliveau — Director Environmental Health Strategy Center, USA.

46. Linda S. Birnbaum PhD — Former Director of the National Institute of Environmental Health Sciences (NIEHS) and the National Toxicology Program (NTP), USA.

47. Alycia Halladay PhD — Chief Science Officer, Autism Science Foundation, Adjunct professor at Dept. of Pharmacology and Toxicology, Rutgers University, USA.

48. Beate Ritz MD PhD — Professor of Epidemiology, Environmental Health, and Neurology, UCLA, Los Angeles, USA.

49. Thomas Zoeller PhD — Professor Emeritus, Department Biology, Department University of Massachusetts, Amherst, USA.

50. Ted Schettler MD MPH	Science Director Science and Environmental Health Network, USA.
51. Charlotte Brody	National Coordinator, Healthy Babies Bright Futures Vice President of Health Initiatives, BlueGreen Alliance, USA.
52. Kosuke Inoue MD PhD	Department of Epidemiology, UCLA Fielding School of Public Health, USA.
53. Laura Vandenberg PhD	Associate Professor, Department of Environmental Health Sciences, School of Public Health and Health Sciences, University of Massachusetts, Amherst, USA.
54. Vasilis Vasiliou, PhD	Professor and Chair of the Department of Environmental Health Sciences. Susan Dwight Bliss, Yale School of Medicine, USA.
55. Bruce P. Lanphear MD MPH	Professor of Health Sciences, Child & Family Research Institute, BC Children's Hospital Professor, Faculty of Health Sciences, Simon Fraser University, Vancouver, BC, Canada.
56.Hagai Levine MD MPH	Chairman of Israeli Association of Public Health Physicians, Israel Medical Association, Hebrew University-Hadassah, Israel.
57. Veerle Bergink MD PhD	Professor, Director of Women's Mental Health Program, Department of Psychiatry, Department of Obstetrics, Icahn School of Medicine at Mount Sinai, NY, USA.
58. Sander van den Driesche PhD	Centre for Discovery Brain Sciences, University of Edinburgh, Edinburgh, UK.
59. Louise Ninett Carlsen MD PhD	Danish Headache Center, Department of Neurology, Righospitalet, Copenhagen University Hospital, Copenhagen, Denmark.
60. Lars Bendtsen MD PhD Dr Med	Clinical Associate Professor, Danish Headache Center, Department of Neurology, Righospitalet, Copenhagen University Hospital, Copenhagen, Denmark.

61. Jerry Heindel PhD	Co-director, Healthy Environment and Endocrine Disruptor Strategies, USA.
62. Brigitte Boizet-Bonhoure PhD	Research Group Leader, Institute of Human Genetics, Centre National de la Recherche Scientifique, Université de Montpellier, Montpellier, France.
63. John van den Anker MD PhD	Division of Clinical Pharmacology, Children's National Hospital, Washington, DC, USA, Department of Paediatric Pharmacology and Pharmacometrics, University Children's Hospital Basel, University of Basel, Switzerland.
64. Bjarne Styrishave PhD	Research Group Leader, Associate Professor, Department of Pharmacy, Faculty of Health and Medical Sciences, University of Copenhagen, Denmark.
65. Gilbert Schönfelder MD	Professor of Toxicology, Head of the Unit Toxicology, Institute of Clinical Pharmacology and Toxicology, Charité - Universitätsmedizin Berlin, corporate member of Freie Universität Berlin and Humboldt-Universität zu Berlin, Institute of Clinical Pharmacology and Toxicology, Germany.
66. Anne Marie Vinnggaard PhD	Professor of Molecular Toxicology, National Food Institute, Technical University of Denmark, Denmark.
67. Arthur David PhD	Professor, Univ Rennes, Inserm, Irset (Institut de recherche en santé, environnementet travail) UMR_S 1085, Rennes, France.
68. Richard M. Sharpe PhD	Professor, Group Leader, MRC Centre for Reproductive Health, Univ of Edinburgh, Scotland.

69. Karel Allegaert MD PhD	Pediatrician, neonatologist and clinical pharmacologist Departments of Development and Regeneration, Pharmaceutical and Pharmacological Sciences, KU Leuven, Leuven, Belgium. Department of Pharmacy, Erasmus University Medical Center, Rotterdam, Netherlands.
70. Song Guo MD PhD	Danish Headache Center, Department of Neurology, Righospitalet, Copenhagen University Hospital, Copenhagen, Denmark.
71. Himi Orhan PhD	Senior Researcher, Department of Pharmaceutical Toxicology, Ege University, Bornova, Turkey.
72. Olle Söder MD PhD	Professor, Department of Women's and Children's Health, Karolinska Institutet Stockholm, Sweden.
73. Olwenn Martin PhD	Professor, Brunel University, Centre for Pollution Research and Policy, London England.
74. Sibylle Ermler PhD	Research Fellow, Brunel University, Centre for Pollution Research and Policy, London England.
75. Martin Scholze PhD	Biostatistician, Brunel University, Centre for Pollution Research and Policy, London England.
76. Edwin Routledge PhD	Professor of Molecular Endocrinology, Brunel University, Centre for Pollution Research and Policy, London England.
77. Ditte Jorgensen MD PhD	Center for Fetal Medicine and Pregnancy. Department of Obstetrics, Rigshospitalet, Copenhagen University Hospital Denmark.
78. Catherine Vedel MD PhD	Center for Fetal Medicine and Pregnancy. Department of Obstetrics, Rigshospitalet, Copenhagen University Hospital Denmark.
79. Ann Z. Bauer ScD (co-author)	Department of Public Health, University of Massachusetts School of Health Sciences, Lowell, Massachusetts, USA.
80. Shanna H. Swan PhD (co-author)	Professor, Department of Environmental Medicine and Public Health, Icahn School of Medicine at Mount Sinai,New York City, New York, USA.

81. David Kriebel ScD (co-author)	Professor Emeritus, Department of Public Health, University of Massachusetts School of Health Sciences,Lowell, Massachusetts, USA.
82. Zeyan Liew PhD, MPH (co-author)	Assistant Professor of Epidemiology, Yale Center for Perinatal, Pediatric, and Environmental Epidemiology,Yale School of Public Health, New Haven, USA.
83. Hugh S. Taylor MD (co-author)	Professor and Chair of Obstetrics, Gynecology and Reproductive Sciences, Professor of Molecular, Cellular,and Developmental Biology, Yale School of Medicine; Chief of Obstetrics and Gynecology, Yale-New Haven Hospital, USA.
84. Carl-Gustaf Bornehag PhD (co-author)	Professor and Head of Public Health Sciences, Department of Health Sciences, Karlstad University, Karlstad, Sweden.
85. Anderson M. Andrade PhD (co-author)	Associate Professor, Departamento de Fisiologia, Setor de Ciências Biológicas, Universidade Federal do Paraná(UFPR) Curitiba, Brazil.
86. Jørn Olsen MD PhD (co-author)	Professor Emeritus, Department of Public Health, Aarhus University, Aarhus, Denmark.
87. Rigmor H. Jensen MD Dr Med (co-author)	Professor of Headache and Neurologic Pain, Director of Danish Headache Center, Department of Neurology, Rigshospitalet-Glostrup, University of Copenhagen, Copenhagen, Denmark.
88. Rod T. Mitchell MD PhD (co-author).	Professor of Developmental Endocrinology, MRC Centrefor Reproductive Health, Queens Medical Research Institute, Edinburgh, Scotland.

89. Niels E. Skakkebaek MD Dr Med (co-author)	Professor of Testicular Dysgenesis and Endocrine Disruption, Department of Growth & Reproduction and EDMaRC, Rigshospitalet, University of Copenhagen, Copenhagen, Denmark.
90. Bernard Jégou PhD (co-author)	Professor and Dean for Research of EHESP-School of Public Health, Univ Rennes, Inserm, Irset (Institut de recherche en santé, environnement et travail) UMR_S1085, Rennes, France.
91. David Møbjerg Kristensen PhD (co-author)	Research Group Leader, Associate Professor, Danish Headache Center, Department of Neurology, Rigshospitalet, University of Copenhagen, Copenhagen, Denmark.

Supplementary table 1: Reproduction Epidemiology						
**Author Date**	**Country Cohort**	**Study Years**	**# of children assessed**	**Pregnancy Acetaminophen (APAP) use prevalence**	**Confounding Control**	**Main Findings (Increased Risk ↑, Decreased Risk ↓, No change ↔)**
Rebordosa et al. 2008[1]	Denmark Danish National Birth Cohort	1996-2003	88,142	30% APAP during 1st trimester Maternal Report	Cox & Poisson regression adjusted for indication, parental age, diabetes, epilepsy, obesity, or low fecundity.	**↔ Hypospadias & cryptorchidism.**<br>APAP ever use in 1st trimester & risk of any congenital abnormalities:<br>↔ Any abnormality (HR=1.01, 95% CI 0.94-1.08)<br>↔ Hypospadias (HR=1.01, 95% CI 0.76-1.33)<br>↔ Undescended testis (HR=1.24, 95% CI 0.79-1.94)<br>↑ medial cysts, fistula, sinus (HR=2.15, 95% CI 1.17-3.95)
Jensen et al. 2010[2]	Denmark Danish National Birth Cohort	1996-2002	47,400	47% APAP<br>5% IBU<br>7% ASA<br>Maternal Report	Cox regression adjusted for indication, fertility treatment, congenital malformations, smoking, diabetes, age, occupation, parity, GA, muscles/joint disease.	**↑ Cryptorchidism.**<br>Prenatal APAP use associated with cryptorchidism when used:<br>↑ both 1st & 2nd trimester (HR 1.33, 95% CI 1.00-1.77)<br>↑ > 4 weeks- week #s 8-14 (HR 1.38, 95% CI 1.05-1.83)<br>↔ IBU, ASA not consistently linked.
Feldkamp et al. 2010 [3]	USA National Birth Defects Prevention	1997-2004	16,110- (11610 cases, 4500 controls)	46 % APAP Maternal Report	Logistic regression. Adjusted for maternal age, education, preconception BMI, gestational diabetes, fever, smoking, folic acid, race, parity.	**↔ Hypospadias.**<br>↔ APAP was not associated with increased risk of any birth defects, including hypospadias. Only evaluated exposure during 1st trimester and single ingredient APAP.
Kristensen et al. 2011[4]	Denmark	1997-2001	491	43% APAP<br>56.2 % Any Analgesic Maternal Report	Logistic regression. Adjusted for indication, other medications, GA, twin, BW, maternal age, smoking, chronic & infectious disease.	**↑Cryptorchidism.**<br>↑ 2+ weeks APAP (OR=2.78, 95% CI 1.13-6.84)<br>↑ > 1 analgesic (ASA, APAP IBU) (OR=7.55, 95% CI 1.94-29.3)<br>↑ >1 analgesic, 2nd trimester (OR=16.1, 95% CI 3.29-78.6)<br>↑ 2+ analgesics, 2+ weeks (OR=21.7, 95% CI 1.83-258)<br>↔ No significant associations to hypospadias, APAP by tri.

Supplementary table 1: Reproduction Epidemiology						
**Author Date**	**Country Cohort**	**Study Years**	**# of children assessed**	**Pregnancy Acetaminophen (APAP) use prevalence**	**Confounding Control**	**Main Findings (Increased Risk ↑, Decreased Risk ↓, No change ↔)**
Kristensen et al. 2011[4]	Finland	1997-1999	1,463	42.3% APAP Maternal Report	Logistic regression. Adjusted for indication, other medications, GA age, twin, BW, maternal age, smoking, chronic & infectious disease.	**↔ Cryptorchidism.**<br>↔ >2 weeks APAP use (OR=0.64, 95% CI 0.15-2.79)<br>↔ No significant association any analgesics.
Wagner-Mahler et al. 2011[5]	France	2002-2005	283-95 Cases, 188 Controls	18.5% APAP and/or ASA Maternal Report	Mann-Whitney Kruskall-Wallis, X2, Fisher's tests and Spearman's tests. Matching on place & date of birth, GA, BW, parent birth country.	**↔ Cryptorchidism.**<br>↔Prenatal APAP and/or ASA not associated w/ Cryptorchidism.
Snijder et al. 2012[6]	Netherlands The Generation R study	2002-2006	3,184	29.9% (75% APAP, 13% NSAID, 12% ASA) Maternal Report	Logistic regression adjusted for indication, co-medication, age, education, country of origin, parity, underlying diseases, folic acid, smoking, alcohol, BMI.	**↑Cryptorchidism.**<br>↑ APAP gestation weeks 14-22 (OR=1.89, 95% CI 1.01-3.51)<br>↑ Any analgesics weeks 14-22 (OR=2.12, 95% CI 1.17-3.83)<br>↔Hypospadias risk- No associations-but few cases (n = 22)
Lind et al. 2013 [7]	USA National Birth Defects Prevention	1997-2007	5,851	23.4% IBU59.3% APAP4.8% ASA Maternal Report	Logistic regression adjusted for maternal age, race, education, pre-pregnancy BMI, previous live births, maternal sub-fertility, study site and year.	**↔ Hypospadias.**
Fisher et al. 2016[8]	United Kingdom Cambridge Baby Growth Study	2001-2009	681 males 401 females	35.6% APAP Maternal Report	Linear Mixed Models. Adjusted for BMI, size. Cohort was representative in terms of maternal age, pre-pregnancy BMI, deprivation index, parity & ethnicity but not smoking.	**↑ Shorter Anogenital Distance (AGD).**<br>↑ APAP exposure during 8-14 weeks of gestation associated with shorter AGD (by 0.27 SD, 95% CI 0.06-0.48) from birth to 24 months of age.<br>↔ Not significant for females or other time points.<br>↔ No association to penile length or testicular descent

Supplementary table 1: Reproduction Epidemiology						
**Author Date**	**Country Cohort**	**Study Years**	**# of children assessed**	**Pregnancy Acetaminophen (APAP) use prevalence**	**Confounding Control**	**Main Findings (Increased Risk ↑, Decreased Risk ↓, No change ↔)**
Smarr et al. 2016[9]	USA	2005-2009	501	93-100% detection of APAP in maternal & paternal urine samples	Cox's proportional odds models for discrete survival time estimated fecundability odds ratios (FORs) adjusted for age, BMI, race, SES, smoking, urinary creatine, chronic conditions, hypothyroidism, hypertension, & maternal urinary APAP.	**↑Increased time to pregnancy (TTP) from paternal APAP exposure.** Higher paternal urinary APAP concentrations were associated with a longer TTP:<br>↑ Highest quartile vs. lowest [FOR=0.65; 95% CI0.45- 0.94)<br>↔ No association w/ continuously models maternal or paternal urinary APAP or p-aminophenol
Lind DV et al. 2017[10]	Denmark Odense Child Cohort	2010-2012	1,027	39.9% (APAP) 4.4% (NSAIDs) 3.3% (APAP + NSAIDs) Maternal Report	Multivariable linear regression adjusted for age, weight. Participants were representative in terms of parity, pre-term birth, smoking, fertility treatment & BMI. NOT representative for education & ethnicity.	**↑ Reduced AGD in male infants (3 months).** Maternal use of both APAP and NSAIDs during the first 28 weeks of gestation:<br>↑ Shorter AGD in males (AGD -4.1 mm, 95% CI -6.4;-1.7)<br>↔ No association in females
Smarr et al. 2017[11]	USA	2005-2009	501	93-100% detection of APAP in paternal urine samples	Linear mixed-effects models adjusted creatinine and age.	**↑ Reduced Sperm motility & Increased DNA fragmentation from paternal APAP exposure.** A 1-standard deviation(SD) increase in log-transformed male urinary APAP associated with:<br>↑Beat cross frequency (β=-0.59 Hz, 95% CI -1.16 - -0.03)<br>↑DNA fragmentation (β= 0.05%, 95% CI 0.01 - 0.09)<br>↑ metabolite p-aminophenol, predominantly associated w/sperm head morphometry

**Supplementary table 1: Reproduction Epidemiology**						
**Author Date**	**Country Cohort**	**Study Years**	**# of children assessed**	**Pregnancy Acetaminophen (APAP) use prevalence**	**Confounding Control**	**Main Findings (Increased Risk ↑, Decreased Risk ↓, No change ↔)**
Interrante et al. 2017[12]	USA National Birth Defects Prevention Study	1997-2001	40,040-29078 cases, 10962 controls	58% only APAP, 70% of NSAID users also used APAP Maternal Report	Logistic regression adjusted for indication, diabetes, high BP, seizures, chronic diseases, injuries, surgeries, age, race, BMI, previous live birth, education, folic acid, alcohol, smoking, antibiotics.	**↔ Hypospadias.**<br>Elevated risk from NSAID use compared to APAP:<br>↑ All NSAIDS preconception-1st tri (OR=1.3, 95% CI 1.1-1.5)<br>↑ Ibuprofen (OR=1.3, 95% CI 1.1-1.5)<br>↔ Aspirin (OR=1.3, 95% CI 0.9-1.7)<br>↔ naproxen (OR= 1.0, 95% CI 0.7-1.6)
Ernst et al. 2019[13]	Denmark Puberty Cohort nested in DNBC	2012-2017	15,822	54% APAP Maternal Report	Regression models adjusted for maternal BMI, alcohol, smoking, TTP, SES, age & age at menarche, fever, infection, inflammation, muscle/ joint disease, parity.	**↑Early Female Pubertal development.**<br>↑Female earlier attainment pubertal development w/ increasing # of weeks of APAP exposure<br>(1.5-3 months earlier pubic hair, axillary hair, & acne development for those prenatally exposed > 12 weeks).<br>↔ Male pubertal development had no strong association.

Time to Pregnancy (TTP), Trimester (TRI), Blood Pressure (BP)

**Supplementary table 2: Reproduction Experimental Studies**				
(1) ***in vivo* studies**				
**Author**	**Model**	**Dose**	**Age, route, and timing of exposure**	**Main Findings (increased ↑, decreased ↓, no change ↔)**
Reel et al. 1992[14]	Male and female mice (continuous breeding protocol)	357, 715, or 1430 mg/kg/day	14 weeks dietary exposure of male:female pairs (during cohabitation).	↓ **number of litters** per pair at the highest dose.
Wiger et al. 1995 [15]	Male mice	400 mg/kg	Adult rats treated for 5 days by intraperitoneal injection	↓ **DNA synthesis in the testes;**<br>↓ **testicular weight** 27 and 33 days after last exposure;<br>↑ **frequency of sperm with changes chromatin structure;**<br>**Delayed spermiogenesis**.

**Supplementary table 2: Reproduction Experimental Studies**				
(1) ***in vivo* studies**				
**Author**	**Model**	**Dose**	**Age, route, and timing of exposure**	**Main Findings (increased ↑, decreased ↓, no change ↔)**
Ratnasooriya et al. 2000 [16]	Male rats	500 or 1000 mg/kg/day	Adult rats treated for 30 days by oral gavage	↓ **fertility;**<br>↓**pregnancy rate per mating;**<br>↓ **sperm count;**<br>↑ **preimplantation embryonic loss.** All these effects were reversible.
Yano, Dolder 2002[17]	Male rats	4.4 mmol/kg	Adult male rats treated with 1 single dose by oral gavage	↑ **Alterations in testicular structure**.
Kristensen et al. 2011[4]	Pregnant Wistar rats	150, 250 or 350 mg/kg/day	13-21 days post coitum (DPC) by oral gavage	↓ **anogenital distance (AGD)** in male fetuses for all three dose groups.
Axelstad et al. 2014[18]	Wister rats exposed to endocrine disrupting chemical mixture*	APAP-360 g/kg/day<br>Other chemicals-100 ,200 ,and 450xs	APAP by oral gavage DPC 13-19 & PND 14-22<br>Other chemicals-DPC 7-21 & PND 1-22	↓ **APAP decreased levator ani/bulbocavernosus muscle (LABC) weights PD55**<br>↑ **APAP increased nipple retention**
Holm et al. 2015[19]	Pregnant C57BL/6JBom mice	50 or 150 mg/kg/day	DPC 7 until birth by oral gavage	↓ **AGD in male offspring** in at the highest dose group.
van den Driesche et al. 2015[20]	Pregnant Wistar rats	APAP-350 mg/kg/day	APAP by oral gavage DPC 13.5-21.5	↓ **AGD in male offspring**
Mandrup et al. 2015[21]	Pregnant Wistar rats exposed to endocrine disrupting chemical (EDC) mixtures*	APAP-360 g/kg/day<br>Other chemical-doses to represent 100, 200, and 450xs	APAP by oral gavage DPC 13-19 & PND 14-22<br>Other chemicals-DPC 7-21 & PND 1-22	↑ **APAP histological changes in adult male mammary glands** similar to A-mix<br>↑**APAP increased male nipple retention**<br>↑ **APAP hypertrophic alveolar epithelium**<br>↑ **APAP increased male duct hyperplasia**<br>↔ APAP did not affect outgrowth or maturation of mammary glands in prepubertal males or females

**Supplementary table 2: Reproduction Experimental Studies**				
(1) ***in vivo*** **studies**				
**Author**	**Model**	**Dose**	**Age, route, and timing of exposure**	**Main Findings (increased ↑, decreased ↓, no change ↔)**
Boberg et al. 2015[22]	Pregnant Wistar rats exposed to endocrine disrupting (EDC) mixtures*	APAP-360 g/kg/day Other chemicals- 100 ,200 ,and 450xs	APAP by oral gavage DPC 13-19 & PND 14-22 Other chemicals- DPC 7-21 & PND 1-22	↓**APAP minor effects on prostate morphology**<br>↓**APAP reduced mRNA level ofPbpc3 in young adulthood** (AAMIX did not).<br>↓Slight reduction in prostate weight changes (similar to Emix)
Holm et al. 2016[23]	Pregnant C57BL/6JBom mice	50 or 150 mg/kg/day	DPC 7 until birth by oral gavage	**↓ AGD in female offspring; ↓ follicle reserves; ↓ fertility; blockage of primordial germ cell proliferation**.
Dean et al. 2016 [24]	Pregnant Wistar rats	350 mg/kg/day	13.5-21.5 DPC by oral gavage; culled at 15.5, 16.5,17.5, 18.5 & 21.5 DPC, and postnatal days (PNDs) 25 & 90	**↓ F1 fetal germ cell (GC) number in both sexes** with delayed meiotic entry in oogonia but accelerated GC differentiation in males;<br>**↓ germ cell numbers & ovary weight** in adult F1 females.<br>**↓ ovarian size & markedly reduced follicle numbers** in F2 females;<br>**↔ in male germ cell count and fertility** in adult F1 males;<br>**↔ in F2 male reproductive development.**
Johansson et al. 2016[25]	Pregnant Wistar rats	360 mg/kg/day	13-19 DPC by oral gavage, culled at 22.5 PND and 13 months PND (adults)	**↓ primordial follicle numbers** in prepubertal females exposed in utero;<br>**↔ in secondary, tertiary and atretic follicles.** At 13 months,<br>**↓ ovary weight and mean number of corpora lutea.**<br>**↑ number of ovaries with follicular cysts** (or cyst like structures).
Hay-Schmidt et al. 2017 [26]	Pregnant C57BL/6JBom mice	50 or 150 mg/kg/day	DPC 7 until birth by oral gavage	**impaired sexual behavior** in adult male offspring<br>**↓ neuronal number in the sexually dimorphic nucleus (SDN**) of the hypothalamic preoptic area (POA)<br>**↓ aggressive territorial display.**
Axelstad M, et al. 2018[27]	Pregnant Wister Rats exposed to endocrine disrupting	APAP-350 g/kg/day Other chemicals- 100 ,200 ,and 450xs high-	APAP by oral gavage DPC 13-19 & PND 14-22 Other chemicals- DPC 7-21 & PND 1-22	**↓ APAP reduced adult sperm counts (17-23% lower)**<br>↑ increased prepubescent testis weight<br>Same effects observed for APAP as high dose AA-Mix & AEP-Mix

**Supplementary table 2: Reproduction Experimental Studies**				
(1) ***in vivo*** **studies**				
**Author**	**Model**	**Dose**	**Age, route, and timing of exposure**	**Main Findings (increased ↑, decreased ↓, no change ↔)**
	chemical mixtures*	end human exposure		
Rossitto et al. 2019 [28]	Pregnant CD1 mice Female offspring	APAP only 60 mg/kg/day APAP+IBU 30 mg/kg/d + 15 mg/kg/d	10.5-13.5 DPC by oral gavage	↔ **did not affect female embryonic germ cells** (APAP or IBU alone)<br>↑**Intergenerational female reproduction effects (Both APAP & IBU)**<br>↓**delayed meiosis entry** & progression in F1 embryonic germ cells<br>↓ **follicular activation** in F1 postnatal ovaries<br>↑ **subfertility** in F2 animals<br>↑ **F2 ovarian aging** with abnormal corpus luteum persistence
Rossitto et al. 2019[29]	Pregnant CD1 mice Male offspring	APAP only 60 mg/kg/day APAP+IBU 30 mg/kg/d + 15 mg/kg/d	10.5-13.5 DPC by oral gavage	↓**germ cell proliferation (APAP or IBU alone)**<br>↑**Intergenerational germ-cell effects (APAP and IBU together)**<br>↓ **sperm count** in F0 adult<br>↓ **sperm motility** in F1 offspring (both parents exposed)<br>↑ **subfertility** in F1 (both parents exposed)
Pereira et al. 2020[30]	Pregnant Wistar female rats	350 mg/kg/day	APAP by oral gavage DPC 6 to DPC 21, or DPC 6 to PN 21.	↑ **APAP male volume and length of seminiferous tubules**<br>↑**APAP increased testis weight**<br>↑ **Induced significant differences in copulation behavior**<br>↑ **Increased testosterone levels at PND 120**<br>Impaired sexual hypothalamic differentiation

[1]Supplementary table 2: Reproduction Experimental Studies-continued				
**(2) *in vitro* and xenograft studies**				
**Author**	**Model**	**Dose**	**Age and timing of exposure**	**Main Findings (increased ↑, decreased ↓, no change ↔)**
Kristensen et al. 2011[4]	Organotypic culture - fetal Sprague Dawley rat testis	1 µM	dpc 14.5 and 3 days incubation	**↓ testosterone production; ↓ prostaglandin D2**.
Kristensen et al. 2012 [31]	Organotypic culture - fetal Sprague Dawley rat testis	1 µM	dpc 14.5 and 3 days incubation	**↓ testosterone production; ↓ prostaglandin D2**.
Albert et al. 2013[32]	Organotypic culture - adult human testis & NCI-H295R adrenocortical human cells	10 or 100 nM	Adult testis explants or H295R cells incubated for 24 or 48h	In the organotypic culture: **↓ testosterone production; ↔ INSL3; ↔ inhibin B; ↔ in testis morphology; ↔ in Leydig cell number; ↓ PGD2; ↓ PGE2;** In the H295R cell line: **↓ testosterone production**
Mazaud-Guittot et al. 2013[33]	Organotypic culture - fetal human testis	10 nM	7-12 gestation weeks testis explants incubated for 24-72h	**↓ INSL3 production; ↓ prostaglandin E2; ↔ testosterone production; ↔ on germ cells**
van den Driesche et al. 2015[20]	Xenograft - human fetal testes grafted into host mice	60 mg/kg/day	14-20 weeks human testis grafted into host mice orally treated for 7 days	**↓ testosterone production; ↓ seminal vesicle weight**
Holm et al. 2016[23]	Organotypic culture - fetal C57BL/6JBom mouse ovary	10 and 100µM	12.5 dpc ovaries incubated for 3 days	**↔ gene expression of markers of germ cell numbers, proliferation, meiosis entry or pluripotency.**

[1]**Supplementary table 2: Reproduction Experimental Studies-continued**				
**(2) *in vitro* and xenograft studies**				
**Author**	**Model**	**Dose**	**Age and timing of exposure**	**Main Findings (increased ↑, decreased ↓, no change ↔)**
Holm et al. 2016[23]	Mouse embryonic stem cells (mESC)	50, 100,or 150 µM	mESC cultured for 72h	↓ **number of cells** indicating inhibition of proliferation, no cytotoxic effects
Gaudriault et al. 2017[34]	Organotypic culture - fetal human testis	0.01 - 100 nM	10-12 gestation weeks human testis explants incubated for 72h	**↔ testosterone production;**
Hurtado-Gonzalez 2018[35]	Organotypic culture - fetal human testis	10 µM	8-11 gestation weeks testis explants incubated for 7 days	**↓ gonocyte number**
Hurtado-Gonzalez 2018[35]	Xenograft - human fetal testes grafted into host mice	60 mg/kg/day	14-20 weeks human testis grafted into host mice orally treated for 1 day	**↓ gonocyte number**
Hurtado-Gonzalez et al. 2018[35]	Organotypic culture - fetal human ovary	10 µM	8-11 gestation weeks ovary explants incubated for 7 days	**↓ number and proliferation of undifferentiated germ cells in fetal ovaries**
Manku et al. 2019[36]	Isolated neonatal gonocytes	33 or 132 µM	PND3 gonocytes incubated for 24 or 72h	**↑ gonocyte proliferation; ↓ mRNA levels of COX-2; ↔ in COX-2 protein expression; ↓ PGE2 and PGF2α.**

*endocrine disrupting chemical (EDC) mixtures consist of :

1) APAP alone

2) A-Mix (antiandrogenic mix) of 8 chemicals (di-n-butylphthalate, diethylhexylphthalate, vinclozolin, prochloraz, procymidone, linuron, epoxiconazole, DDE)

3) E-Mix (estrogenic mixture) of 4 chemicals (bisphenol A, 4-methylbenzylidene camphor, 2-ethylhexyl4-methoxycinnamate and butylparaben)

4) AEP-Mix consisting of A-Mix and E-Mix plus APAP

Supplementary table 3: Neurodevelopment Epidemiology
Cohort studies

Author Date	Country Cohort	Study Years	# of children assessed	Prevalence of Pregnancy Acetaminophen (APAP) use	Domain	Confounding Control	Main Findings (Increased Risk ↑, Decreased Risk ↓, No change ↔)
Brandlistuen et al. 2013[37]	Norway Norwegian Mother and Child Cohort Study (MoBA)	1999-2008	48,631	46% Maternal Report	Motor Skills, Adaptive Behavior, Affect, Attention	Sibling control analysis, regression models adjusted for febrile illness, infections, co-medications.	**↑ Adverse Neurodevelopment at 3 years.**<br>Long-term prenatal APAP exposure (>28 days) associated with poorer:<br>↑ Gross motor development (β=0.24, 95% CI 0.12-0.51)<br>↑ Communication (β=0.20, 95% CI 0.01-0.39)<br>↑ Externalizing behavior (β=0.28, 95% CI 0.15-0.42)<br>↑ Internalizing behavior (β=0.14, 95% CI 0.01-0.28)<br>↑ Higher activity levels (β=0.24, 95% CI 0.11-0.38)<br>↔ fine motor, emotionality, sociability, shyness, use of ibuprofen<br>↑Dose-response
Liew et al. 2014 [38]	Denmark Danish National Birth Cohort (DNBC)	1996-2002	64,322	56% Maternal Report	Attention, Executive Function, Adaptive Behavior, Social Communication	Regression models adjusting for maternal musculoskeletal diseases, fever, inflammation or infection, psychiatric illnesses, NSAIDS use.	**↑ ADHD/ hyperkinetic disorder at 7 -11 yrs.**<br>**Prenatal** APAP exposure associated with:<br>↑ ADHD diagnosis (HR=1.37, 95% CI 1.19-1.59)<br>↑ ADHD treatment (HR= 1.29, 95% CI 1.15-1.44)<br>↑ ADHD-like behaviors (RR=1.13, 95% CI 1.01-1.27)<br>↑ Dose-response for all outcomes (P trend < .001)
Thompson et al. 2014[39]	New Zealand Auckland Birthweight Collaborative (ABC)	1995-1997	871	50% Maternal Report	Attention, Executive Function	Regression model adjusted for fever, maternal stress, and psychological conditions.	**↑ ADHD symptoms at 7 & 11 yrs.**<br>Children of mothers who used APAP during pregnancy had:<br>↑ higher total difficulties scores<br>↑ increased risk of ADHD at 7 and 11 years of age<br>↔ No risk differences for anti-inflammatories, aspirin, antibiotics, antacids

Supplementary table 3: Neurodevelopment Epidemiology
Cohort studies

Author Date	Country Cohort	Study Years	# of children assessed	Prevalence of Pregnancy Acetaminophen (APAP) use	Domain	Confounding Control	Main Findings (Increased Risk ↑, Decreased Risk ↓, No change ↔)
Liew et al. 2015[40]	Denmark DNBC	1996-2002	64,322	56% Maternal Report	Attention, Executive Function, Adaptive Behavior, Social Skills, Communication	See Liew et al. 2016	**↑ Autism Spectrum Disorder (ASD) with Hyperkinetic Disorder (HKD) 8-13 yrs.**<br>Any prenatal APAP exposure associated with:<br>↑ ASD w/ HKD symptoms (HR=1.51, 95% CI 1.19-1.92)<br>↔ Other ASD cases (HR=1.06, 95% CI 0.92–1.24)<br>↑ Dose-response ASD w/ HKD (P trend < .001)
Liew et al. 2016[41]	Denmark DNBC	1996-2002	1,491	56% Maternal Report	Attention, Executive Function	Regression models adjusting for maternal musculoskeletal diseases, fever, inflammation or infection, NSAIDS, psychiatric illnesses.	**↑ Subnormal attention & executive function 5 yrs.**<br>Children prenatally exposed to APAP had a higher risk of subnormal:<br>↑ Overall attention (OR=1.5, 95% CI 1.0-2.5)<br>↑ Selective attention (OR=1.5, 95% CI 1.0-2.4)<br>↑ Executive function-parent (OR=1.5, 95% CI 0.9-2.3)<br>↑Dose-response
Liew et al. 2016b[42]	Denmark DNBC	1996-2002	1,491	56% Maternal Report	Cognition	See Liew et al. 2016, also maternal IQ (genetic confounding)	**↑ Lower Intelligence Quotient (IQ) 5 yrs.**<br>Prenatal APAP for use other than fever associated with:<br>↑ Lower Verbal IQ (2.7 points, 95% CI -0.19-5.6)<br>↑ Lower Performance IQ (4.3 points, 95% CI 0.30-8.3)<br>↔ No IQ change when mothers w/ fever used APAP<br>↔ No dose-response observed
Stergiakouli et al. 2016[43] (68)	England Avon Longitudinal Study of Parents & Children (ALSPAC)	1991-1992	7,796	53% Maternal Report	Attention, Executive Function	Negative Control Exposure analysis. Regression methods adjusted for headaches, musculoskeletal issues, infection, genetic ADHD risk, psychiatric illness.	**↑ ADHD symptoms at 7 yrs.** Prenatal APAP exposure at 32 weeks was associated with:<br>↑ Conduct problems (RR=1.42, 95% CI 1.25-1.62)<br>↑ Hyperactivity symptoms (RR=1.31, 95% CI 1.16-1.49)<br>↑ Emotional symptoms (RR=1.29, 95% CI 1.09-1.53)<br>↑ Total difficulties (RR=1.46, 95% CI 1.21-1.77)<br>↔ No association-maternal postnatal or partner's APAP use

Supplementary table 3: Neurodevelopment Epidemiology
Cohort studies

Author Date	Country Cohort	Study Years	# of children assessed	Prevalence of Pregnancy Acetaminophen (APAP) use	Domain	Confounding Control	Main Findings (Increased Risk ↑, Decreased Risk ↓, No change ↔)
Avella-Garcia et al. 2016[44]	Spain Infancia y Medio Ambiente (INMA project)	2004-2008	2,644	43% Maternal Report	Attention, Executive Function	Regression model adjusted for maternal chronic illness, fever, or urinary tract infection.	**↑ Autism Spectrum Disorder (ASD) & Attention function at 1 & 5 yrs.**<br>APAP Exposed offspring had higher risks of:<br>↑ hyperactivity/impulsivity (IRR=1.41, 95% CI 1.01-1.98)<br>↑ K-CPT commission errors (IRR=1.1, 95% CI 1.03–1.17)<br>↑ lower detectability score (β=−0.75, 95%CI−0.13- −0.02)<br>↑Higher CAST scores in males (β = 0.63, 95% CI 0.09-1.18)<br>↑ Dose-response - hyperactivity, CE, Male CAST scores<br>↔ inattention, hit reaction time ↓CAST female<br>↑Dose-response
Vlenterie et al. 2016[45]	Norway MoBA	1999-2008	51,200	41% Maternal Report	Motor Skills, Adaptive Behavior, Affect, Attention	Propensity Score Matching	**↑ Adverse Neurodevelopment at 1.5 years.**<br>Long-term prenatal APAP exposure (>28 days) associated with:<br>↑ Communication problems (OR=1.38, 95% CI 0.98-1.95)<br>↑ Delayed motor milestones (OR=1.35, 95% CI 1.07-1.70)<br>↔ Gross & fine motor, behavioral problems, temperament problems, short term APAP exposure.<br>↑Dose-response, no effects observed w/ short-term use
Bornehag et al. 2017[46]	Sweden Swedish Environmental Longitudinal, Mother child, Asthma/allergy (SELMA)	2007-2010	754	59% maternal report & urine biomarkers (100% had detectable APAP)	Language	Regression methods adjusted for maternal colds, antibiotics, painkillers, asthma meds, NSAIDS.	**↑ Female Language Delays (LD) at 30 months.**<br>APAP use in pregnancy weeks 8-13 were associated with:<br>↑ LD in girls exposed to > 6 APAP vs. 0 tablets (OR=5.92, 95% CI 1.10-31.94)<br>↑ LD in girls w/ mothers' urinary APAP in highest quartile (OR=10.34, 95% CI 1.37-77.86)<br>↔ No association of APAP to LD in boys<br>↑Dose-response in girls

Supplementary table 3: Neurodevelopment Epidemiology
Cohort studies

Author Date	Country Cohort	Study Years	# of children assessed	Prevalence of Pregnancy Acetaminophen (APAP) use	Domain	Confounding Control	Main Findings (Increased Risk ↑, Decreased Risk ↓, No change ↔)
Ystrom et al. 2017[47]	Norway MoBA	1999-2009	112,972	47% Maternal Report	Attention, Executive Function	Regression methods adjusting for familial risk/parent symptoms of ADHD, anxiety, depression, fever, infections, pain conditions.	**↑Attention Deficit Hyperactivity Disorder (ADHD)**<br>Pregnancy APAP exposure & ADHD at 3-15 yrs:<br>↑ 1st trimester (HR=1.07, 95% CI 0.96-1.19)<br>↑ 2nd trimester (HR=1.22, 95% CI 1.07-1.38)<br>↑ All 3 trimesters (HR=1.27, 95% CI 0.99-1.63)<br>↑ >29 days of use (HR=2.20, 95% CI 1.50-3.24)<br>↓ <8 days of use (HR=0.90, 95% CI 0.81-1.00)<br>↑Fever/infection, 22-28 days (HR=6.15, 95% CI 1.71-22.0)<br>↑ Paternal, ↔ Maternal, preconception use of APAP<br>↑Dose-response
Skovlund et al. 2017[48]	Norway MoBA	1999-2008	45,646	46% Maternal Report	Language, Social Communication	Opioid use, work, paternal education, BMI, parity, smoking, benzodiazepine, selective serotonin reuptake inhibitor pregnancy use	**↑ Poorer Communication skills**<br>↑ Dose-response by increasing periods of APAP Use. All 3 time periods (OR= 1.17 95% CI 1.06–1.30)<br>↔ language competency- APAP use<br>↔ communication skills & language competency-opioids
Gervin et al. 2017[49]	Norway MoBA subset Epigenome association study	1999-2008	384	Cases: 100% APAP biomarkers detected in cord blood / Controls 0%.	Attention, Executive Function	DNA methylation analysis. Gene ontology analysis. Surrogate variable analyses suggest that there are no unmeasured covariates with a strong confounding effects.	**↑DNA Methylation changes in ADHD by APAP.**<br>↑ Individuals with ADHD and exposed long term (> 20 days) to APAP during development had significantly different DNA methylation compared to controls (no APAP, No ADHD) at genes involved in oxidative stress, neural transmission, and olfactory sensory pathways .<br>↑ Neither APAP nor ADHD alone were associated with DNA methylation differences.<br>↑ Dose-response, methylation differences <20 & >20 days

**Supplementary table 3: Neurodevelopment Epidemiology**<br>**Cohort studies**							
**Author Date**	**Country Cohort**	**Study Years**	**# of children assessed**	**Prevalence of Pregnancy Acetaminophen (APAP) use**	**Domain**	**Confounding Control**	**Main Findings (Increased Risk ↑, Decreased Risk ↓, No change ↔)**
Tovo-Rodrigues et al. 2018[50]	Brazil 2004 Pelotas Birth Cohort	2004	3,566	28% Maternal Report	Attention, Executive Function	Regression models adjusting for maternal infection, NSAID use, self-reported depression, anxiety, and treatment.	**↑ Emotional & hyperactivity problems at 6 & 11 yr**<br>At 6 yrs prenatal APAP increased boys odds of:<br>↑ Emotional problems (OR=1.47 95% CI 1.07-2.02)<br>↑ Hyperactivity/inattention (OR=1.42 95% CI 1.06-1.92)<br>At 11 yrs prenatal APAP increased boys odds of:<br>↑ Emotional problems (OR=1.31 95% CI 0.99-1.73)<br>↑hyperactivity/inattention (OR=1.25 95% CI 0.95-1.65)<br>↔ No associations for girls
Petersen et al. 2018 [51]	Denmark DNBC	1996-2002	185,617	49% Maternal report	Brain structure /Neurology, Motor Skills, Cognition, Language	See Liew et al. 2016	**↑ Cerebral Palsy (CP).**<br>Prenatal APAP exposure ever associated w/ increased risk of:<br>↑ Overall CP (OR=1.3, 95% CI 1.0-1.7)<br>↑ Unilateral spastic CP (OR=1.5, 95% CI 1.0-2.2)<br>Aspirin exposure ever in pregnancy associated with:<br>↑ Bilateral spastic CP (OR=2.4, 95% CI 1.1-5.3)<br>↑Dose-response
Ji et al. 2018[50]	USA Boston Birth Cohort (BBC)	1998-2016	1,180	55% had a detectable APAP biomarkers in plasma sampled 1-3 days post-partum	Attention, Executive Function	Regression models adjusted for maternal fever, intrauterine infection or inflammation, breastfeeding.	**↑ ADHD physician diagnosis median age 7 yrs.**<br>ADHD risk from APAP exposure-biomarkers 3rd vs. 1st tertile:<br>↑ Unchanged APAP (OR=2.05, 95% CI 1.27-3.32)<br>↑ N-acytl-l-cystein-S-yl (OR=2.03, 95% CI 1.26-3.27)<br>↑ APAP glucuronide (OR=2.00, 95% CI 1.26-3.18) ↔<br>No association to ASD, other developmental disabilities<br>↑ Effect modification by intrauterine infection/inflammation<br>↑ Dose-response specific to ADHD

Supplementary table 3: Neurodevelopment Epidemiology
Cohort studies

Author Date	Country Cohort	Study Years	# of children assessed	Prevalence of Pregnancy Acetaminophen (APAP) use	Domain	Confounding Control	Main Findings (Increased Risk ↑, Decreased Risk ↓, No change ↔)
Laue et al. 2018[52]	Canada GESTation and the Environment (GESTE) Cohort	2007-2009	118	53% had detectable APAP biomarkers in meconium	Cognition	Regression methods adjusting age, BMI, maternal intelligence, parity, SES, child characteristics.	**↔ Neurocognitive development (IQ) at age 6 to 8.**<br>In utero APAP exposure, as measured in meconium, was not statistically significantly associated with decreased scores on any subset of the Weschler Intelligence Scale for Children.<br>↔ No dose-response detected
Ruisch et al 2018[53]	England ALSPAC	1991-1992	6,300 Maternal rating 4,400 Teacher rating	53% Maternal Report	Adaptive Behavior, Social Skills or Social Communication	Regression models adjusted for comorbid ADHD, ODD or CD, infections, genetic risk, drug use, life stress, anxiety & depression.	**↑ Oppositional-defiant (ODD) and conduct disorders (CD) at 7 & 9 yrs.**<br>Prenatal APAP use associated with:<br>↑ teacher rating ODD (IRR=1.24 98.3% CI 1.05-1.47)<br>↑ Also associated - life stress, smoking, depression<br>↔ Not associated - maternal infection, aspirin, alcohol
Rifas-Shiman et al. 2019[54]	USA Project Viva	1992-2002	1,225	46.1% Mothers reported using >10 doses, 65.3% of infants given > 6 doses Maternal Report	Executive Function	Maternal antibiotic use, depression, anti-depressants. Infant respiratory tract infection. Home emotional support & cognitive stimulation.	**↑Subnormal Executive Function (EF) & behavior 8 yrs.**<br>Prenatal exposure associated with lower EF measure:<br>↑High APAP use (β=1.64 pts, 95% CI 0.59,2.68)<br>↑Any Ibuprofen Use (β=1.56 pts, 95% CI 0.19,2.92)<br>Infant exposure (high) associated w/ lower EF measure:<br>↑APAP (β=1.69 pts, 95% CI 0.51,2.87)<br>↑Ibuprofen (β=1.40 pts, 95% CI 0.25,2.55)<br>↑Dose-response
Leppert et al. 2019 [55]	England Avon Longitudinal Study of Parents & Children(ALSPAC)	1990 - 2018	7,921	53.7% early 42.8% late pregnancy Maternal Report	Attention, Executive Function	Polygenic risk scores.	**↔ APAP association may be due to genetic confounding.**<br>↑ Exposure to APAP during pregnancy associated with ADHD (RR=1.45, 95% CI 1.18-1.78)<br>↑ ADHD maternal polygenic risk score (PRS) associated with late pregnancy APAP use (OR=1.11, 95% CI 1.04-

Supplementary table 3: Neurodevelopment Epidemiology
Cohort studies

Author Date	Country Cohort	Study Years	# of children assessed	Prevalence of Pregnancy Acetaminophen (APAP) use	Domain	Confounding Control	Main Findings (Increased Risk ↑, Decreased Risk ↓, No change ↔)
							1.18)
Tronnes et al. 2019[56]	Norway MoBA	1999-2008	32,934	45.9% ever, 1 trimester 25.4% 2 tri 15.1%, 3 tri 5.4% Maternal Report	Adaptive Behavior, Social Skills or Social Communication	Linear & Generalized linear models, Propensity Scores (PS) methods. Covariates include maternal health conditions, anxiety, depression, other meds, alcohol, BMI.	**↑Adverse Neurodevelopmental Outcomes at age 5.**<br>↔ Sensitivity analyses suggest unmeasured confounders may have biased estimates.<br>Prolonged prenatal APAP was associated with childhood:<br>↑Shyness at 5 yrs (β=-0.62, 95% CI-1.05, -0.19)<br>↑Internalizing Behavior (RR=1.36, 95% CI 1.02, 1.80)<br>↑Externalizing behavior (RR=1.22, 95% CI 0.93, 1.60)<br>↔ Communication problems<br>↑Dose-response
Ji et al. 2019[57]	USA BBC	1998-2018	996	Placenta Cord Blood APAP metabolites - Unchanged APAP detectable in 100%	Attention, Executive Function, Adaptive Behavior, Social Skills, or Social Communication	Regression models adjusted for pregnancy stress, depression, anxiety, intrauterine infection, fever. Delivery type, preterm birth, maternal ADHD.	**↑ADHD and ↑Autism in childhood.**<br>Risk by APAP exposure-total burden biomarker vs. 1st tertile:<br>↑ ADHD 2nd tertile (OR=2.26, 95% CI 1.40-3.69)<br>↑ ADHD 3rd tertile (OR=2.86, 95% CI 1.77-4.67)<br>↑ ASD 2nd tertile (OR=2.14, 95% CI 0.93-5.13)<br>↑ ASD 3rd tertile (OR=3.62, 95% CI 1.62-8.60)<br>↑ Dose-response both ADHD & ASD

**Supplementary table 3: Neurodevelopment Epidemiology**<br>**Cohort studies**							
**Author Date**	**Country Cohort**	**Study Years**	**# of children assessed**	**Prevalence of Pregnancy Acetaminophen (APAP) use**	**Domain**	**Confounding Control**	**Main Findings (Increased Risk ↑, Decreased Risk ↓, No change ↔)**
Liew et al. 2019[58]	USA<br>Nurses' Health Study II	1993-2005	8,856	14% used regularly during pregnancy Maternal Report	Attention, Executive Function	Negative control exposure analysis to control for genetics & maternal chronic disease. Depression, arthritis, migraine, aspirin & NSAIDS.	**↑ ADHD Maternal report.**<br>Prenatal APAP use associated with childhood:<br>↑ ADHD (OR=1.34 95% CI 1.05-1.72)<br>↑ Pregnancy subset (OR=1.46 95% CI 1.01-2.09)<br>↔ No association to 4 years prior & post pregnancy APAP exposure.
Golding et al. 2019[59]	England<br>ALSPAC	1991-1992	14,062	43.9% Maternal Report	Attention, Executive Function	Regression analysis. Exposome analysis-hypothesis free selection of 15 variables associated w/ taking APAP during gestation weeks 18 to 32 in final model.	**↑ADHD & related behaviors & outcomes.**<br>↑ APAP exposure between 18 & 32 weeks gestation associated 12 outcomes of hyperactive or attention related behaviors in preschoolers.<br>↔ little sign of adverse associations at later ages, w/ exception of males conduct problems until 9 yrs.
Chen et al. 2019 [60]	Taiwan Health Insurance Database	1998-2008	4,750 (950 cases, 3,800 controls)	68% obtained APAP as identified by insurance claims, did not capture APAP use purchased by mother	Attention, Executive Function	Logistic regression models adjusted for demographic characteristics, maternal infections & mental health disorders, comorbid perinatal conditions.	**↑ADHD at 5+ years old.**<br>ADHD risk associated with prenatal APAP exposure in:<br>2nd trimester (OR= 1.19; 95% CI, 1.00-1.40)<br>↑ 1st & 2nd trimesters (OR = 1.28; 95% CI, 1.00-1.64)<br>↑ Any trimester (OR = 1.20; 95% CI, 1.01-1.42)<br>↔ No dose response identified.<br>APAP exposure from prescription records, also available OTC

Supplementary table 3: Neurodevelopment Epidemiology
Cohort studies

Author Date	Country Cohort	Study Years	# of children assessed	Prevalence of Pregnancy Acetaminophen (APAP) use	Domain	Confounding Control	Main Findings (Increased Risk ↑, Decreased Risk ↓, No change ↔)
Bertoldi et al. 2020[61]	Brazil 2015 Pelotas Birth Cohort	1992-2002	3,818	Maternal Report 57.6% during 1st or 2nd trimester	Motor Skills, Cognition, Language	Maternal age, pre-pregnancy BMI, education, parity, race/ethnicity. Smoking, alcohol, depression, antibiotic, ibuprofen use household income, child sex.	**↓ Adverse Neurodevelopment at 2 years.**<br>INTERGROWTH-21st Neurodevelopment Assessment 1st & 2nd tri exposure<br>↓Higher total score (β 0.08 95% CI 0.01 -0.16)<br>↔ When stratified-null results boys,↓ protective effect girls only
Bertoldi et al. 2020[61]	USA Project Viva	1992-2002	1217	Maternal Report 69.9% during 1st or 2nd trimester	Motor Skills, Cognition, Language	Maternal age, pre-pregnancy BMI, education, parity, race/ethnicity. Smoking, alcohol, depression, antibiotic, ibuprofen use, household income, child's sex.	**↑ Adverse Neurodevelopment at 3 years.**<br>Wide Range Achievement of Visual Motor abilities (WRAVMA) 1st & 2nd tri exposure:<br>↑ lower drawing score (β -1.51 95% CI -2.92 --0.10)<br>↔ pegboard, matching or total score<br>↔ Peabody Picture Vocabulary Test 1st & 2nd tri exposure<br>↔ No association to any outcome for infant 1st yr exposure
Tovo-Rodrigues et al. 2020[62]	Brazil 2004 Pelotas Birth Cohort	2004	3624	28% Perinatal Maternal Report	Adaptive Behavior, Social Skills or Social Communication	Poisson and linear regression adjusted for family and maternal sociodemographic and health factors, medication use, child sex.	**↔ Low neurodevelopmental performance 24 months**<br>↔ Emotional or Behavioral problems at 48 months

**Supplementary table 3: Neurodevelopment Epidemiology**<br>**Cohort studies**							
**Author Date**	**Country Cohort**	**Study Years**	**# of children assessed**	**Prevalence of Pregnancy Acetaminophen (APAP) use**	**Domain**	**Confounding Control**	**Main Findings (Increased Risk ↑, Decreased Risk ↓, No change ↔)**
Baker et al. 2020 [63]	Quebec, Canada Sherbrooke Gestation and Environment Cohort	2007-2009	345	57.7% had APAP detected in meconium samples	Attention, Executive Function	Inverse probability weighting with propensity scores w/ child sex & maternal covariates. Evaluated ADHD in mothers & use of APAP at delivery in sensitivity analyses.	**↑ADHD at 6-7 years old.**<br>↑ ADHD Any vs. no APAP (OR=2.43, 95% CI 1.41-4.21)<br>↑ Dose response-each 2xs APAP increase ADHD odds 10%<br>↑MRI assessment - APAP exposed showed increased negative connectivity between frontoparietal & default mode network to sensorimotor cortices.<br>↑Function brain connectivity changes mediated an indirect effect on hyperactivity.

**Supplementary table 3: Neurodevelopment Epidemiology**<br>**Other Epidemiologic Studies**							
**Author Date**	**Country Cohort**	**Study Years**	**# of children assessed**	**Prevalence of Pregnancy Acetaminophen (APAP) use**	**Domain**	**Confounding Control**	**Main Findings (Increased Risk ↑, Decreased Risk ↓, No change ↔)**
Schultz et al. 2008 [64]	Case-control study of infant APAP exposure in relation to ASD	2005-2006	83 cases 80 control	N/A	Attention, Executive Function	Age, gender, mother's ethnicity, illness concurrent with measles-mumps-rubella vaccination	**↑ASD under 5 years**<br>APAP after MMR vaccination associated with:<br>↑ ASD (OR 6.11, 95% CI 1.42—26.3)<br>↑ ASD (OR 3.97, 95% CI 1.11—14.3) children w/ regression in development only<br>↑ ASD (OR 8.23, 95% CI 1.56—43.3) children w/ post-vaccination sequelae only

Bauer and Kriebel 2013[65]	Ecological study APAP usage in relation to ASD	1984-2005	N/A	up to 65%	Attention, Executive Function	None	**↑ASD prevalence correlation**<br>↑ Prenatal use of APAP correlated with country-level autism/ASD prevalence (r = 0.80).<br>↑ Male country-level autism/ASD prevalence correlated w/ country's circumcision rate (r = 0.98) (circumcision proxy for newborn male APAP exposure)
Parker et al. 2019[66]	Canada & USA Reanalysis of Case-control craniofacial malformations	1996-2002	560	60% Maternal report on use thru pregnancy month 5 . Interviewed ~1 yr after delivery	Adaptive Behavior, Affect, Attention	Headache, fever & respiratory infection w/o fever, allergy. Depression & anxiety.	**↔ Behavioral Problems at 6-12 years.**<br>↔ Total behavioral problems-maternal report (mean difference 0.1, 95% CI -2.1, 2.3)<br>↔ Clinical Behavioral Problems-maternal report (RR 1.33, 95% CI 0.67, 2.58)
**Randomized Control Trial**							
Juujärvi S et al. 2019[67]	Finland - Randomized, double-blind APAP use for ductus arteriosus closure	2013-2014	44 very preterm babies	Randomized to intravenous APAP or a placebo for four days	Motor Skills, Adaptive Behavior, Affect, Attention, Cognition	Not Applicable	**↔ Adverse Neurodevelopment at 2 years.**<br>↔ Neurodevelopmental stages, as quantified by the Griffiths test, were similar for those exposed & unexposed to early APAP.<br>↔ No signs of autism reported.

Supplementary table 3: Neurodevelopment Epidemiology
Other Human Studies

Author Date	Age	Study Design	Main Findings (Increased Risk ↑, Decreased Risk ↓, No change ↔)
Jetten et al. 2012[68]	adults age 48+/-4.5	Evaluated the effects on metabolite formation and global gene-expression changes including full-genome human miRNA expression changes in blood/urine samples when given 0.5, 2 or 4 gms APAP every 6 hours over 24 hrs,	↑ Omics methods identified low therapeutic level APAP doses **trigger an immune response (2 gm) and oxidative stress-related gene response (4 gm)**

Cohen et al. 2018[69]	Adults	Established the human metabolome profile of metabolites that characterizes use of APAP. Used genomics to elucidate the mechanisms targeted by APAP.	**↑ APAP impact on hormone homeostasis.** Identified a distinctive pattern ↓ sulfated sex hormones with use of APAP -- very low levels of neurosteroids such as pregnenolone sulfate, DHAS and androstenediol. Effect on sex hormones ~ 35 yrs of aging.
Wright et al. 2019[70]	Children 0-9 yrs	Evaluated cerebellar tissue of 58 post-mortem children to determine if experienced inflammation at time of death.	**↑ Suggest a sensitive period to inflammation in the human cerebellum begins at about 1 yr based on post-mortem cerebellar tissue**. ↑in the messenger RNA (mRNA) of the COX-1 and COX-2 enzymes, strongly correlated with mRNA levels of aromatase. Autism related etiology, suggests APAP potential factor.
Addo et al. 2020 [71]	Newborn (placenta)	Evaluated effect of APAP on mRNA expression, protein expression and hormone synthesis in human placental JEG-3 cells. Treatments ranged from 0.1 - 5mM APAP.	**↑ APAP influenced hormone synthesis & protein expression**.<br>At high doses influenced gene expression:<br>↓Reduced- aromatase (CYP19A1)<br>↓Reduced Type 1 HSD3B1<br>↑Increased HSD17B1<br>↓Reduced aromatase (CYP19A1) protein expression. ↓Significant dose-dependent decrease in estradiol secretion.

*All adjusted for age, smoking, education

Acetaminophen (APAP), Ibuprofen (IBU), Aspiring (ASA), Body Mass Index (BMI), Socioeconomic Status (SES), Gestational Age (GA), Birthweight (BW)
Time to Pregnancy (TTP), Trimester (TRI), Blood Pressure (BP)

Supplementary table 4: Neurotoxicity
Experimental Studies

(1) *in vivo* studies				
**Author**	**Model**	**Dose**	**Age, route, and timing of exposure**	**Main Findings (increased ↑, decreased ↓, no change ↔)**
Posadas et al. 2010[72]	Adult Sprague Dawley female rats	250 or 500 mg/kg/day	Single intraperitoneal doses and sample collection 1h, 3h or 6h after administration	↑ **Neuronal death** at doses that do not cause hepatotoxicity
Leroux et al. 2010[73]	Neonatal mice	10, 100 mg/day	Treatment with ibotenate to induce excitotoxic brain injury day 5, APAP post (day 5-9) or pre-treatment (day 1-5)	↓**Decreased white matter lesions on day 10 by post-treatment APAP**<br>↔ no difference in white matter lesions by pre-treatment APAP
da Silva et al. 2012[74]	Adult male mice	600mg/kg/day	single intraperitoneal administration; brain samples collected 4h after exposure	**Marked neurotoxicity** with changes in mitochondrial functioning;<br>↑ **ROS and oxidative stress**<br>↓ **glutathione.**
Gould et al. 2012[75]	Adult mice (two inbred strains with inherently low sociability, BTBR and 129S1/SvImJ)	APAP 1, 10, 100, and 400 mg/kg/day	single intraperitoneal administrations 50 minutes prior to behavioral testing.	↑ **Social interaction** in adult male mice.
Dean et al. 2012[76]	neonatal rats	40 mg/kg/day	PND 7-13 by subcutaneous injection	↑ **Increase in the spinophilin content in the cerebellar posterior vermis, a marker of dendritic spine number in the cerebellum.**
Blecharz-Klin et al. 2013 [77]	Adult male mice	10 and 50 mg/kg/day	3 months-old rats received daily subcutaneous injections of APAP for 8 weeks	**Modulation of** serotonergic and noradrenaline **neurotransmission** in prefrontal cortex, hypothalamus, striatum. Subtle **changes in working memory & behavior.**
Blecharz-Klin et al. 2013[77]	Adult male mice	10 and 50 mg/kg/day	3 months-old rats received daily subcutaneous injections of APAP for 8 weeks	↓ **Levels of amino acids in the striatum** (glutamine, glutamic acid, taurine, alanine, aspartic acid) **and hypothalamus** (glycine); ↑ **γ-aminobutyric acid (GABA)** in the prefrontal cortex.

Supplementary table 4: Neurotoxicity
Experimental Studies

(1) *in vivo* studies				
**Author**	**Model**	**Dose**	**Age, route, and timing of exposure**	**Main Findings (increased ↑, decreased ↓, no change ↔)**
Yisarakun et al. 2014[78]	Adult male rats	200 mg/kg	Acute: one single dose<br>Chronic: 1 dose/day for 30 days orally.	**Damage to the cerebral micro vessels/ microvasculature; ↑ protein expression of cell adhesion molecule ICAM-1; brain ultrastructural alterations.**
Viberg et al. 2014[79]	Neonatal mice	30 or 60 (2x 30) mg/kg/day	Single dose or two doses (4h apart) on postnatal day (PND) 10 by subcutaneous injection.	Highest dose (2x 30 mg/kg) induced **long-lasting effects on cognitive function at adulthood** & altered adult response to APAP.<br>**↑ BNDF levels in neonatal frontal cortex; ↓ BNDF in neonatal parietal cortex; ↔ BNDF in neonatal hippocampus**
Lichtensteiger et al. 2015[80]	Pregnant Wister Rats exposed to EDC mixtures*	APAP-360 g/kg/day<br>Other chemicals- 100 ,200 ,and 450xs	APAP administered by oral gavage during gestation days (GD) 13-19<br>Other chemicals- GD day 13-21 & PND days 1-22	**↑Effects of APAP on developing rat brain regions and**<br>**↑ Genes related to glutamatergic synapses, GABAergic neurons & autism spectrum disorders**<br>↑APAP effects almost identical to AEP-Mix<br>↑ APAP antiandrogenic effects on male reproductive endpoints.
Thiele et al. 2015[81]	Pregnant mice	50 or 250 mg/kg/day	single administration on dpc 12.5. Tissue samples 1 or 4 days after dosing	**↑ Frequency of mature dendritic cells & regulatory T-cells at 250 mg/kg; ↓plasma progesterone; ↓ fetal weight & hematopoietic stem cells.**<br>Impaired maternal immune system, endocrine adaptation to pregnancy, and placental function; impaired fetal maturity & immune development.
Saad et al. 2016[82]	Pregnant CD1 mice	150 mg/kg/day	day post coitum (DPC) 7 until delivery.	**↔ No significant differences in vertical (rearing), horizontal, or total locomotor activity**<br>↔ no differences found in volumes of 29 brain areas on MRI or in neuronal quantifications
Zhao et al. 2017[83]	Adult male mice	100 mg/kg/day	APAP by intraperitoneal injections 0.5 h prior to intracerebroventricular administration of LPS or saline. APAP treatment daily up to 3 days.	**↓APAP *neuroprotective effect.*** APAP attenuates lipopolysaccharide (LPS) induced cognitive impairment by antioxidant action of suppression of proinflammatory cytokines and microglia activation induced by LPS in the hippocampus

Supplementary table 4: Neurotoxicity
Experimental Studies

(1) *in vivo* studies				
**Author**	**Model**	**Dose**	**Age, route, and timing of exposure**	**Main Findings (increased ↑, decreased ↓, no change ↔)**
Blecharz-Klin et al. 2017[84]	Continuous exposure of rats from pregnancy to adult life	5 or 15 mg/kg/day	Daily from beginning of pregnancy until postnatal week 8 through drinking water	M**ajor changes in serotonergic & dopaminergic neurotransmission** in the prefrontal cortex & striatum at 8 weeks; **major change in the amino acid level in hippocampus & cortex; Behavioral changes in cognitive performance & visuomotor skills at 8 weeks.**
Philippot et al. 2017[85]	Neonatal mice	60 (2x 30) mg/kg/day	single dose or two doses (4h apart) on PND 3, 10 or 19 by subcutaneous injection.	Adverse effects on **adult behavior and cognitive function** in male and female mice exposed on PND 3 and 10 but not on PND 19.
Blecharz-Klin et al. 2018[86]	Continuous exposure of rats from pregnancy to adult life	5 or 15 mg/kg/day	Daily from beginning of pregnancy until postnatal week 8 through drinking water	**↓ Striatal BDNF; changes in social behavior & exploration at eight weeks.**
Philippot et al. 2018[87]	neonatal mice	30 or 60 (2x 30) mg/kg/day (isolated or combined w CB1R agonist (WIN)	single dose or two doses (4h apart) on PND 10 by subcutaneous injection.	**↓ Locomotion, rearing and total activity in adult mice** neonatally exposed to APAP (2 x 30 mg/kg) or co-exposed to APAP and WIN. Co-exposed mice displayed significant **lack of habituation**, when compared to controls & single agent exposure.
Kandis et al. 2018[88]	Adult male rats	100, 200 or 400 mg/kg/day	oral gavage for 11 days; empathy test 30 min after administration, every other day.	**↓ Empathy-like behavior** after a single high dose or repeated low dose administrations in correlation with ↓ **oxytocin & vasopressin levels** in the prefrontal cortex & amygdala.
Chen et al. 2018[89]	Adult male rats	50 or 300 mg/kg/day	7-10 weeks-old rats treated with a single intraperitoneal dose 90 minutes prior to behavioral testing (experimental days 1 & 3)	**↑ Anxiety-like and anhedonic behavior**, and **impaired recognition memory** at highest APAP dose

**Supplementary table 4: Neurotoxicity**<br>**Experimental Studies**				
(1) *in vivo* studies				
**Author**	**Model**	**Dose**	**Age, route, and timing of exposure**	**Main Findings**<br>**(increased ↑, decreased ↓, no change ↔)**
Saeedan et al. 2018[90]	Albino rat pups	50 mg/kg	Fever induced (by LPS or vaccines) and APAP subcutaneous 50 mg/kg on PND 5	**↑ Social behavioral changes correlating with pro-inflammatory markers**<br>↓ locomotion<br>↓ negative geotaxis<br>↓ olfactory discrimination<br>↑ exploration, ↓nest seeking<br>↑ thermal nociception time<br>↑ oxidative and inflammatory markers.
Klein et al. 2019[91]	Pregnant rats	350 mg/kg/day	day post coitum (DPC) 6 until delivery.	**↑ Neurofunctional Alterations** -impaired nest seeking behavior; **↑ apomorphine-induced behavioral stereotypy; ↓ rostral grooming**. **Olfactory losses & ↑ locomotion** in females; **↔ glutathione (GSH), BDNF levels, or lipid hydroperoxide** in the prefrontal cortex or hippocampus in prepubertal rats
Blecharz-Klin et al. 2019[92]	Continuous exposure of rats from pregnancy to adult life	5 or 15 mg/kg/day	Daily from beginning of pregnancy until postnatal week 8 via drinking water	**↓ hypothalamic concentration of glutamic acid** at APAP lowest dose; **changes on dopaminergic and noradrenergic neurotransmission.**
Lalert et al. 2019[93]	Adult male rats	200 mg/kg bw	acute, 15 days or 30 days oral administration	**↔ after acute (24 h) exposure;**<br>**↑** Long-term APAP treatment induced **synaptic degeneration in the hippocampus & frontal cortex.** Synapses from 15-day treatment rats showed a marked shortening of active zones & widening of the synaptic cleft with decrement of SYP and PSD-95 proteins. With 30-day APAP treatment, the **alteration of the synaptic ultrastructure & proteins** more evident along with **oxidative stress**, the depletion of GSH & elevation of protein carbonyl oxidation (PCO) levels.
Vigo et al. 2019[94]	Adult male rats	1000 mg/kg/day	one single intraperitoneal dose; analyses after 24 hrs	Neurotoxic effects and hepatotoxicity, but not acute liver failure;<br>**↓ locomotion; ↓brain dopamine levels; ↑ oxidative stress; reactive astrogliosis.**

Supplementary table 4: Neurotoxicity Experimental Studies

(1) *in vivo* studies				
**Author**	**Model**	**Dose**	**Age, route, and timing of exposure**	**Main Findings (increased ↑, decreased ↓, no change ↔)**
Motawi et al. 2019[95]	Adult male rats	400 mg/kg /day	oral administration for 28 days.	**↓ Thyroid axis hormones; ↓ brain neurotransmitters & ↓ serum testosterone; ↑ oxidative stress & brain DNA damage; histopathological changes in the brain.**
Koehn et al. 2019[96]	Pregnant rats; newborn rats; adult female rats	15 mg/kg/day	Intraperitoneal and/or intravenous administration. Acute: single 15 mg/kg dose; Chronic: 2x/day 15mg/kg for 5 days to fetuses, newborn, and adult rats.	Acute & Chronic APAP treatment resulted in higher amounts in the fetal brain and cerebrospinal fluid (CFS) than in adult. When chronically treated, **100% of APAP in the fetal plasma was identified in the fetal brain & CSF suggesting there was no restriction on APAP entry.** Barrier efflux capacity may have been exceeded. The placenta was able to partly impede passage.

Supplementary table 4: Neurotoxicity Experimental Studies-continued

**(2) *in vitro* studies**				
**Author**	**Model**	**Concentration**	**timing/duration of exposure**	**Main Findings (increased ↑, decreased ↓, no change ↔)**
Posadas et al. 2010 [72]	Primary culture of rat brain cortical neurons	0.5, 1 or 2 mM	cells incubated for up to 24 h	**Neuronal death** associated with mitochondrial–mediated mechanisms that involve cytochrome c release and caspase 3 activation; **↑ CYP2E1 activity and protein expression**
Schultz et al. 2012[97]	Cortical neuronal cell culture of developing mice	10-250 mg/ml, p-aminophenol 1-100 mg/ml	cortical neurons from embryonic day 15 mice incubated in vitro for 24h	**↔ in neurons exposed to APAP**; **↓ cortical neurons viability after incubation with APAP metabolite p-aminophenol.**
Tantarungsee et al. 2018[98]	Mouse astrocyte cells (C8-D1A)	100 μM	in vitro exposure for 24 h, 16 days or 28 days	**↔ after acute (24 h) exposure;** Chronic treatment (16 & 28 days) **↑ protein expression of proinflammatory cytokines Il-1β and TNF-α; activation of NF-kB**
Vigo et al. 2019[94]	Primary culture of newborn rats astrocytes	1, 5, 10, or 20 mM	cells incubated for 48h	**↓cell viability at 10 and 20 nM; reactive astrogliosis.**

*Exposed to 4 endocrine disrupting mixtures:

1) APAP alone

**Supplementary table 4: Neurotoxicity<br>Experimental Studies**				
		(1) *in vivo* studies		
**Author**	**Model**	**Dose**	**Age, route, and timing of exposure**	**Main Findings (increased ↑, decreased ↓, no change ↔)**

2) A-Mix (antiandrogenic mixture) of 8 chemicals - di-n-butyl phthalate (DBP), di-(2-ethylhexyl) phthalate (DEHP), vinclozolin, prochloraz, procymidone, linuron, epoxiconazole, dichlorodiphenyl-dichloroethylene (p,p'-DDE)

3) E-Mix (estrogenic mixture) of 4 chemicals-octyl methoxycinnamate, 4-methylbenzylidene camphor, bisphenol A, butyl paraben

4) AEP-Mix consisting of A-Mix and E-Mix plus APAP

Supplementary table 5:
Reviews

**Author**	**Review year**	**Neurological studies reviewed or referenced**		**Reproduction -Urogenital studies reviewed or referenced**		**Scope and Review Method**	**Results**	**Main Conclusions**	**Identified Strengths**	**Identified Weaknesses**
		**Epi**	**Lab**	**Epi**	**Lab**					
Bauer et al. (this paper)	2020	37 total, 29 cohort studies	28	14	30	Narrative review of reproductive, urogenital, and neurological effects of APAP from both human and experimental studies.	Large majority of studies suggest APAP has endocrine disrupting properties and exposure both prenatal and perinatal, may increase risk of adverse neurological, urogenital, and reproductive outcomes.	**The current evidence is sufficient to warrant precautionary action.** We recognize the limitations of the existing data and call for a focused research effort. Potential risks should be communicated. APAP use by pregnant women should occur only under the advice of their health care provider and at the lowest effective dose for the shortest possible time.	Inclusion of all available APAP neurologic and reproductive studies, both human and experimental from 1/1/1995-10/25/2020. Call for research prioritization to overcome limitations of existing studies recognizing the public health implications may be substantial as APAP is so commonly used.	Need further understand of biologic mechanisms. Need epidemiologic studies designed specifically to; (*i*) reduce confounding by genetic factors and indication for use, (*ii*) minimize exposure and outcome misclassification by upgrading standard targeted biomonitoring methods with additional APAP metabolites that extent the window of assessment; and (*iii*) examine postpartum exposure.

Supplementary table 5:
Reviews

Author	Review year	Neurological studies reviewed or referenced		Reproduction -Urogenital studies reviewed or referenced		Scope and Review Method	Results	Main Conclusions	Identified Strengths	Identified Weaknesses
		Epi	Lab	Epi	Lab					
Kim et al.[99]	2020	35 total ADHD risk factors studies.	0	0	0	Umbrella review of systematic reviews with meta-analyses of observational studies that examined associations of potential environmental risk factors with diagnosis of ADHD.	Evidence was convincing (Class I) for maternal APAP exposure during pregnancy (RR 1·25, 95% CI 1·17 to 1·34), maternal pre-pregnancy obesity, childhood eczema, hypertensive disorders during pregnancy, and pre-eclampsia.	**Evidence of association to ADHD was convincing (class I) for maternal APAP exposure during pregnancy**	95% CI, heterogeneity I2 statistic = 26%, No indication of Large heterogeneity, small study effect, excess significance bias, or loss of significance under 10% credibility ceiling. Meta-analyses assessed with AMSTAR 2 (A Measurement Tool to Assess Systematic Reviews) as high when protocol assessment was ruled out.	Excluded studies without data for reanalysis
Masarwa et al. [100]	2020	8	0	0	0	Meta-analysis and bias analysis of association to ADHD epidemiology. Assessment of the role of unmeasured confounding, selection bias and exposure misclassification.	When adjusted estimates were pooled across all studies, the risk ratio (RR) for ADHD was 1.35 (95% confidence interval [CI] 1.25, 1.46; I 2 = 48%).	**Our ability to conclude a causal association is limited.** Bias analysis suggests that the previously reported association between acetaminophen use during pregnancy and an increased risk of ADHD in the offspring may be due to unmeasured confounding.	Use of random effects models	Sensitivity analysis for unmeasured confounding in this meta-analysis showed that a confounder of 1.69 on the RR scale would reduce to 10% the proportion of studies with a true effect size of RR >1.10. The observed association could be confounded by parental ADHD.

Supplementary table 5: Reviews

Author	Review year	Neurological studies reviewed or referenced		Reproduction -Urogenital studies reviewed or referenced		Scope and Review Method	Results	Main Conclusions	Identified Strengths	Identified Weaknesses
		Epi	Lab	Epi	Lab					
Talge[101]	2020	6	0	0	0	Narrative Review of Prenatal APAP and Neurodevelopment Epidemiology.	5 of the 6 studies that included childhood assessment data reported links between self-reported APAP use and at least one aspect of behavioural-level functioning.	**Weight of evidence is inconclusive regarding a possible connection between APAP use in pregnancy and ADHD in children.**	Despite marked variability in approach these studies represent a comprehensive epidemiologic investigation of the topic, as they featured a range of study designs , informants, assessment ages (2-16 years), functional domains, objectives (confounder identification and effect estimation), and settings.	Heterogeneity of numerous aspects of study designs. Limited coherence of the reported neurobehavioral difficulties. Concerns about residual confounding by indication, familial confounding and bias from parent reported exposure and outcomes. Small effect sizes.
Parker et al.[66]	2019	6 New studies in special issue	0	0	0	Narrative editorial accompanying special issue on neurodevelopment and APAP in Pediatric and Perinatal Epidemiology	Issue studies observed positive associations with some neurodevelopmental outcomes, as have been reported in previous literature. However, questions regarding the validity of these observed associations surface as the overarching theme- systematic error, confounding, exposure, and outcome misclassification.	**" Understanding the degree to which these associations still persist after accounting for potential sources of bias simultaneously will be paramount in painting a clearer picture of APAP and neurodevelopment."**	An assortment of analytical tools were utilized to assess the validity of findings. These methods including bias analyses, negative controls, sensitivity analysis, and comparison of outcome assessments by mother and teacher.	No discussion of the biologic plausibility of observations. No studies used biomarkers of exposure.

Supplementary table 5: Reviews

Author	Review year	Neurological studies reviewed or referenced		Reproduction-Urogenital studies reviewed or referenced		Scope and Review Method	Results	Main Conclusions	Identified Strengths	Identified Weaknesses
		Epi	Lab	Epi	Lab					
Gou et al. [102]	2019	8	3	0	0	Meta-analysis of neurodevelopmental epidemiology of ADHD in the offspring of women exposed to APAP during pregnancy.	8 cohort studies with a total of 244,940 participants included. The pooled adjusted risk ratio was 1.25 (95% CI= [1.17, 1.34]). 3rd trimester exposure (risk ratio: 1.26; 95% CI = [1.08, 1.47]). 28 days or longer exposure (risk ratio: 1.63; 95% CI= [1.23, 2.16]).	**There is an association between maternal acetaminophen use during pregnancy and the risk of attention deficit/hyperactivity disorder in offspring.** The timing and duration of acetaminophen use during pregnancy may have a major effect on the risk of attention deficit/hyperactivity disorder.	All studies except the oldest found a statistically significant association. Studies were prospective cohort studies, which may reduce recall bias and avoid reverse causality. Exposure misclassification would be non-differential which usually leads to underestimation of risk. Dose-response seen for duration of use. Association differed by trimester of maternal use.	Results were susceptible to misclassification of exposure, outcomes, and potential confounding by indication for use or other unmeasured factors.
Black et al. [103]	2019	6	0	0	0	Narrative review of multiple medications for pain management in pregnancy.	Emerging evidence points to possible adverse effects on infant neurodevelopment with prenatal APAP use. The proposed biological explanation for this is disruption of the maternal endocrine system, which regulates fetal brain development.	**Overall, the evidence does not provide definitive conclusions about an association between APAP use.** APAP remains the drug of choice for pregnant women who require a simple analgesic. The dose should not exceed the recommended 4 g/day and ideally use should not be prolonged.	Large, prospective cohort studies have reported an association between APAP use in pregnancy and attention deficit hyperactivity disorder (ADHD) symptoms in offspring, with larger associations observed with increased frequency and duration of exposure.	High risk for residual confounding, selection bias and potentially genetic confounding.

**Supplementary table 5: Reviews**											
**Author**	**Review year**	**Neurological studies reviewed or referenced**		**Reproduction -Urogenital studies reviewed or referenced**		**Scope and Review Method**	**Results**	**Main Conclusions**	**Identified Strengths**	**Identified Weaknesses**
		**Epi**	**Lab**	**Epi**	**Lab**					
Bauer et al.[104]	2018	9	21	2	9	Narrative review of epidemiology and experimental studies of APAP and neurodevelopment. Data pooling of human studies was viewed as not appropriate due to heterogeneity in outcomes.	9 of 9 human prospective cohort studies suggest a statistically significant increased risk of adverse neurodevelopmental outcomes following prenatal APAP exposure.	**Pregnant women should be cautioned against indiscriminate use of APAP. Further studies are urgently needed with; precise indication of use and exposure assessment of use both in utero and in early life**.	Studies were prospective cohort studies, which may reduce recall bias and avoid reverse causality. Exposure misclassification would be non-differential which usually leads to underestimation of risk. Dose-response relationship identified and trimester effects with late second to 3rd trimester use identified as higher risk.	Risk for residual confounding both genetic and by indication. Potential selection bias and exposure misclassification.
Masarwa et al.[105]	2018	7	3	1	2	Meta-analysis of neurodevelopmental epidemiology of ADHD and autistic spectrum disorder (ASD) in the offspring of women exposed to APAP during pregnancy.	The pooled risk ratio for ADHD was 1.34 (95% CI: 1.21, 1.47; I2 = 72%), ASD 1.19 (95% CI: 1.14, 1.25; I2 = 14%), and hyperactivity symptoms 1.24 (95% CI: 1.04, 1.43; I2 = 93%).	**Findings are concerning; however, results should be interpreted with caution given that the available evidence consists of observational studies and is susceptible to bias.**	Retrospective cohorts included 132,738 mother-child pairs, with follow-up periods from 3 to 11 years. Random effects methods for pooled results. Sensitivity analysis showed no change in strength of association when studies were excluded with a long duration of exposure and exceptional study design. Conducted meta-regression.	Studies differed widely in exposure and outcome assessment. Observational studies susceptible to several potential sources of bias.

Supplementary table 5: Reviews

Author	Review year	Neurological studies reviewed or referenced		Reproduction -Urogenital studies reviewed or referenced		Scope and Review Method	Results	Main Conclusions	Identified Strengths	Identified Weaknesses
		Epi	Lab	Epi	Lab					
Arendrup et al. [106]	2018	0	0	0	10 total	Narrative review of experimental studies of pregnancy APAP and female reproductive development.	All studies suggest that prenatal exposure to APAP may disrupt female development. APAP decreased primordial follicle pools and reduced fertility. Rodent studies suggest that a sensitivity window may exist comparable with the last weeks of 1st trimester during human pregnancy. The phenotypes observed resemble premature ovarian insufficiency syndrome in humans.	**We highlight an urgent need for more studies to verify these data including both experimental and epidemiological approaches.**	Similar phenotypes and effects were observed by 3 independent research teams and in two different species of rodents strengthens the notion of a possible cause-and-effect relation. A possible mechanism or mode of action of APAP could be as a disruptor of mitosis early in germ cell development.	Effect timing differed by experiment. The limited number of animals utilized. Effects may not be transferable to humans. Some dosages were higher than human equivalents.
Mc Crae et al.[107]	2018	4	1	4	6	Narrative review summarizing the current evidence on the harms associated with chronic APAP use, focusing on cardiovascular disease, asthma, renal injury, as well as neurodevelopmental, endocrine, and reproductive effects of in utero exposure.	Extrapolation of preclinical toxicology data to humans may suggest associations with asthma, ADHD, and androgen disruption.	**Practical advice would be to avoid the protracted use of APAP for nonfebrile illness. Only weak associations between APAP exposure and neurodevelopmental issues have been identified, and no causal link can be inferred.** Preclinical toxicology data may suggest associations with asthma, ADHD, and androgen disruption.	Extrapolation of preclinical toxicology data to humans may suggest associations with asthma, ADHD, and androgen disruption.	The epidemiological studies may be subject to confounding by unmeasured environmental factors, recall bias, diagnostic inaccuracy (most rely on coding data or parental recall for their outcomes) and differences in drop-out rates. Notably, few studies confirm the effect of duration and timing of APAP.

Supplementary table 5: Reviews

Author	Review year	Neurological studies reviewed or referenced		Reproduction -Urogenital studies reviewed or referenced		Scope and Review Method	Results	Main Conclusions	Identified Strengths	Identified Weaknesses
		Epi	Lab	Epi	Lab					
Drobnis and Nangia [108]	2017	0	0	3	8	Narrative review of pain medications and male reproduction.	APAP has been shown to cause sperm abnormalities, including DNA fragmentation, and to increase time to pregnancy and may prove to be of greater concern. In rodents, APAP has negative impacts on seminiferous tubule histology and fertility.	**There is evidence that APAP decreases human male fertility, and this is also seen in rodents.** This direct effect on male fertility is highly concerning. Recommending discontinuation of this commonly used medication by men attempting fertility would be prudent as we await further information on pregnancy outcomes and offspring health.	The effects of NSAID administration on reproductive hormones have proved to be modest or absent. In contrast APAP has been shown in humans to have endocrine disrupting activity on the developing male fetus and to affect adult male fertility.	N/A
Kilcoyne & Mitchell [109]	2017	0	0	9	9	Narrative review assessing the impact of in-utero exposure to APAP on male reproductive development	Epidemiological studies demonstrate associations with cryptorchidism in the offspring, primarily in relation to APAP exposures during the second trimester and prolonged duration of exposure, while experimental studies suggest that APAP can reduce testosterone production by the human fetal testis.	**Based on the current evidence discussed, it cannot be concluded that exposure to APAP is a direct cause of male reproductive disorders nor that analgesics should simply be avoided during pregnancy.** A pragmatic approach is to ensure that where analgesics are deemed to be necessary, that they are used at the minimum therapeutic dose for the shortest possible duration. Further study needed including postnatal exposure.	Experimental evidence of APAP exposure reducing testosterone was shown to manifest in subsequent disorders with the epidemiology suggesting APAP associations to cryptorchidism and reduced sperm production/counts.	Epidemiological studies inconsistent and based on self-reported analgesic use which can be subject to recall bias. In-vitro culture conditions cannot replicate normal human testosterone production or the in-vivo environment. Both the in-vitro and xenograft systems lack a feto-placental unit, although APAP has been demonstrated to cross the placenta and enter the fetal circulation in similar concentrations to maternal plasma.

Supplementary table 5: Reviews										
**Author**	**Review year**	**Neurological studies reviewed or referenced**		**Reproduction -Urogenital studies reviewed or referenced**		**Scope and Review Method**	**Results**	**Main Conclusions**	**Identified Strengths**	**Identified Weaknesses**
		**Epi**	**Lab**	**Epi**	**Lab**					
Toda [110]	2017	13		2	0	Narrative Review of Prenatal APAP and risks to the fetus.	Prenatal APAP was associated with asthma, lower performance intelligence quotient (IQ), shorter male infant anogenital distance, ASD, gross motor development, communication issues, ADHD, poorer attention and executive function, and behavioral problems in childhood. Long-term use and/or a lot of usage likely increases adverse effect occurrence.	**APAP is not Safe in Pregnancy. In order to protect the fetus, I would like public organizations or academic associations to declare danger (or safety) of acetaminophen in pregnancy. We should recognize risks of APAP. APAP should be used at the lowest effective dosage and for the shortest time.** APAP should not be withheld from children or pregnant women for fears it might develop adverse effects.	Each article has poor power to show risks of APAP, however, the integration of the articles that showed adverse effects of APAP may have power to show them.	At the present moment, no public organizations or academic associations have declared the danger of APAP in pregnancy.
Allegaert and van de Anker[111]	2017	8	3	2	0	Narrative review of different aspects of pharmacokinetics, efficacy, and safety of APAP during pregnancy and in neonates.	Prenatal APAP exposure is widespread (37-53%). There are associations with several neuro-behavioral outcome variables with high numbers needed to harm (48-250). Animal studies support a link to neuro-behavioral outcomes. Epi studies provide evidence for a cryptorchidism or hypospadias association. Proposed mechanism is impaired masculinization by reduced fetal testicular testosterone production.	**Long-term safety following neonatal exposure to APAP remain limited, and reports are based on epidemiological association-type studies. These associations suggested between fetal exposure and subsequent risks for atopy, fertility, or neurobehavioral problems**. Therefore, it is worth developing long-term safety studies, looking specifically at these aspects following neonatal exposure.	8 out of 8 cohort studies of prenatal APAP in relation to neurobehavioral outcomes suggest an association.	Exposure to APAP and outcome variables are regularly based on maternal recall, interviews, or questionnaires subject to recall bias. There is a limited set of potential covariates available to build the predictive models. Experimental studies have identified a link between fetal APAP exposure and neurobehavioral outcome but remain difficult to translate to humans.

Supplementary table 5: Reviews											
**Author**	**Review year**	**Neurological studies reviewed or referenced**		**Reproduction -Urogenital studies reviewed or referenced**		**Scope and Review Method**	**Results**	**Main Conclusions**	**Identified Strengths**	**Identified Weaknesses**
		**Epi**	**Lab**	**Epi**	**Lab**					
Society of Maternal and Fetal Medicine (SMFM)[112]	2017	5	0	0		Narrative review of recent observational studies reporting an association between prenatal APAP and increased risk for adverse neurological outcomes in childhood. Aim is to provide guidance to practicing obstetric care providers as they discuss these issues with their patients.	All 6 studies reported an association, albeit weak.	**Based on our evaluation, we believe that the weight of evidence is inconclusive regarding a possible causal relationship between APAP use and neurobehavioral disorders in the offspring.** Communication regarding the risks versus benefits should occur. SMFM continues to advise that APAP be considered a reasonable and appropriate medication choice for the treatment of pain and/or fever during pregnancy.	3 were considered large studies.	The definition and diagnosis of these illnesses, especially ADHD, continues to evolve and as causes are unknown may be subject to both unknown environmental & genetic confounding. Studies have significant methodological limitations including maternal self-report of APAP, lack of dose quantification, & outcome measurement using questionnaire, potential recall bias, interview bias, confounding and failure to adjust for multiple testing.

Supplementary table 5:
Reviews

Author	Review year	Neurological studies reviewed or referenced		Reproduction -Urogenital studies reviewed or referenced		Scope and Review Method	Results	Main Conclusions	Identified Strengths	Identified Weaknesses
		Epi	Lab	Epi	Lab					
Parker et al. [113]	2017	8			0	Narrative Review and Hypothesis paper	The data presented suggests one explanation for the increased prevalence of autism is that increased exposure to APAP, exacerbated by inflammation and oxidative stress, is neurotoxic in babies and small children.	**The use of APAP in babies and young children may be much more strongly associated with autism than its use during pregnancy**, perhaps because of well-known deficiencies in the metabolic breakdown of pharmaceuticals during early development. Research into long-term effects of infant APAP, particularly shortly after birth, is urgent.	Hundreds of studies describing the epidemiology of autism and the numerous and varied risk factors for autism have a straightforward explanation: autism could be an APAP-induced brain injury facilitated by oxidative stress and inflammation in newborns and young children. This hypothesis satisfies Occam's razor, it is intuitive and accounts for observations.	No high-quality cohort studies have investigated the association of APAP use by babies and autism.
Andrade[114]	2016	5	7	0	0	Narrative Review of use of APAP during pregnancy and the risk of ADHD in the offspring.	Several prospective studies have found an association between gestational APAP exposure and ADHD-like behaviors, use of ADHD medication, and ADHD diagnoses in offspring during childhood; the only negative study was a small investigation that examined only one aspect of attention as an outcome.	**APAP may not be as safe in pregnancy as is widely believed.** As fever during pregnancy may be associated with adverse gestational outcomes, given the present level of uncertainty, until more data are available, the use of APAP in pregnancy should not be denied in situations in which the need for the drug is clear.	Most of the studies adjusted analyses for many (but not all) confounds associated with ADHD risk. Additionally, a large population study is cited suggesting no association between fever and infection and ADHD arguing against confounding by indication. The risk may be dose dependent. Many theoretical biologic mechanisms acknowledged.	Potential confounds, including genetic risk and postnatal variables, may not have been comprehensively identified, measured, and adjusted for in all studies. Some studies did not utilize a formal diagnosis of ADHD, and analyses were not corrected for multiple hypothesis testing.

**Supplementary table 5: Reviews**											
**Author**	**Review year**	**Neurological studies reviewed or referenced**		**Reproduction -Urogenital studies reviewed or referenced**		**Scope and Review Method**	**Results**	**Main Conclusions**	**Identified Strengths**	**Identified Weaknesses**
		**Epi**	**Lab**	**Epi**	**Lab**					
Andrade[115]	2016	4	0	0	0	Narrative review of use of APAP during pregnancy and the risk of ASD	In 2 studies, population-level use of APAP was associated with trends in the incidence of ASD. In 1 cohort study, gestational exposure to APAP was linked to poorer neurodevelopment & hyperactivity, but not to social and emotional deficits. In 1 cohort study, gestational exposure to APAP was associated with an increased risk of ASD only with hyperkinetic disorder; displaying a dose-response association.	**The limited evidence available at present does not support the conjecture that use of APAP during pregnancy contributes to the suggested secular increase in autism incidence.**	Confounding by indication may not be the explanation for findings, as risk elevation specific to APAP (no association found to Ibuprofen) and findings were similar when analyses were restricted to women who did not experience infection or fever during pregnancy.	A limitation of the 2 ecological studies APAP use in the population may have merely been a marker for an unmeasured ASD risk factor. In the light of existing data associating APAP use during pregnancy and subsequent risk of ADHD, these finding may suggest that the predisposition, if any, is toward the hyperkinetic syndrome rather than to autism.
U.S. Food and Drug Administra-tion (FDA) [116]	2015	3	0	0	1	Narrative Review of epidemiologic relationship between prenatal APAP and relation to ADHD. Critical review of 1 study (Liew et al. 2014). Comment on Brandlistuen et al. 2013 and Streissguth et al. 1987.	Liew et al. 2014:<br>↑ ADHD/ hyperkinetic disorder at 7 -11 yrs.<br>Prenatal APAP exposure associated with:<br>↑ ADHD diagnosis<br>↑ ADHD treatment<br>↑ ADHD-like behaviors<br>↑ Dose-response for all outcomes (P trend < .001).<br>Streissguth et al. 1987: conflicting	**Based on our evaluation of these studies, we believe that the weight of evidence is inconclusive regarding a possible connection between APAP use in pregnancy and ADHD in children.**	Large study included 64,322 pregnancies to assess ADHD medication use, and 40,916 pregnancies to assess child behavior.<br>Identified dose-response -associations for all outcomes were strongest for APAP use in multiple trimesters and for more than 20 weeks during pregnancy.	The authors did not assess overall markers of health, including health care utilization and/or medication utilization in the year prior to and during the index pregnancy. No information was provided on the acetaminophen strength and number of dosage units taken, no ability to assess a dose-response relationship. Did not assess with clinical ADHD diagnoses.

Supplementary table 5: Reviews

Author	Review year	Neurological studies reviewed or referenced		Reproduction -Urogenital studies reviewed or referenced		Scope and Review Method	Results	Main Conclusions	Identified Strengths	Identified Weaknesses
		Epi	Lab	Epi	Lab					
Amino-shariae [117]	2015	2	1	2	0	Narrative Review of new issues related to safety, labeling, dosing, and a better understanding of the analgesic effect of APAP.	Large-scale epidemiologic studies report evidence for second-generation adverse effects of APAP. Prenatal exposure to APAP is associated with neurodevelopmental and behavioral disorders. APAP reported a hormone disrupter (i.e., it interferes with sex and thyroid hormone function essential for normal brain development).	**May not be considered a safe drug during pregnancy. Available evidence suggests that indiscriminate usage of this drug is not warranted and its administration to a pregnant patient should be considered with great caution.**	Brandlistuen et al. utilization of sibling control design.	N/A
Hoover et al. [118]	2015	4	0	0	0	Narrative review of epidemiologic relationship between prenatal APAP and relation to ADHD.	4 studies examining the effects of prenatal acetaminophen exposure on subsequent ADHD behaviors were identified. Of these, only one early study (1987) found no link to ADHD behaviors while the other studies found statistically significant correlations.	**While there does appear to be a mild correlation, current data do not provide sufficient evidence that prenatal APAP exposure leads to development of ADHD symptoms late in life.** The significant limitations should not preclude the prenatal use of APAP for fever and pain relief. APAP should be used at the lowest effective dose for the shortest duration of therapy and while weighing the risks, benefits. Educating expectant mothers and health care providers on using APAP appropriately should be a priority.	Liew et al. 2014 examined a large yet homogenous cohort and collected reliable ADHD assessment data. APAP exposure was associated with higher risk of 3 different measures of ADHD- having a hospital diagnosis of hyperkinetic disorder, using ADHD medications, and exhibiting ADHD-like behaviors at 7 years. The association appeared to be dose and duration dependent.	The diagnostic approach to ADHD is changing and the etiology is unclear. Studies may have significant confounding and bias.

Supplementary table 5: Reviews

Author	Review year	Neurological studies reviewed or referenced		Reproduction -Urogenital studies reviewed or referenced		Scope and Review Method	Results	Main Conclusions	Identified Strengths	Identified Weaknesses
		Epi	Lab	Epi	Lab					
Brune et al. [119]	2015	2	0	2	1	Narrative review of history and safety of APAP.	Studies suggest APAP may cause acute liver failure, kidney damage and development of the fetus and the newborn child. Male children born from mothers taking APAP during pregnancy are more prone to suffer from cryptorchidism as compared to boys without exposure to the drug.	**'Today APAP would (and should) not be admitted to human use'. It appears timely to reassess the risk/benefit ratio of this compound.** APAP has second-generation problems due that may cause not only acute liver failure and kidney damage but also impaired development of the fetus and the newborn child.	Specificity of APAP on neurologic effects identified (associations not found to ibuprofen)	The argument that most of these epidemiologic studies are not prospective, controlled, and blinded are correct. In addition, they lack information on the dose and duration of exposure. RCTs, as much as we would like to have them, are not feasible for ethical reasons.
De Fays et al.[120]	2015	4	10	0	0	Narrative review of prenatal APAP and neurodevelopment Epidemiology	Exposure to APAP in utero was associated with a moderate (if not weak) statistically significant increased risk of ADHD/HKD disorders or neurodevelopmental detrimental effects for the children. In the 2 larger studies, prolonged use resulted in stronger associations.	**Limitations in the studies' design prevent inference on a causal association with ADHD/HKD or child neurological development.** We conclude that additional well-designed cohort studies are necessary to confirm or disprove the association. In the context of current knowledge, APAP is still to be considered safe in pregnancy and should remain the first-line treatment for pain and fever.	Recent animal data showed that cognition and behaviour may be altered following exposure to therapeutic doses of APAP in early development. However, no firm conclusion can be made on the relevance to humans. Specificity to APAP (no association to ibuprofen) in the epi studies may argue against confounding by indication.	Effects are in the weak to moderate range and a moderate amount of bias or confounding could explain the observed associations.

Supplementary table 5: Reviews										
**Author**	**Review year**	**Neurological studies reviewed or referenced**		**Reproduction -Urogenital studies reviewed or referenced**		**Scope and Review Method**	**Results**	**Main Conclusions**	**Identified Strengths**	**Identified Weaknesses**
		**Epi**	**Lab**	**Epi**	**Lab**					
Tiegs et al.[121]	2014	2	0	2		Narrative review of the epidemiological findings and cellular mechanisms responsible for APAP-mediated prenatal risk for asthma and other second generation APAP effects.	Adverse effects include an increased risk for development of wheezing and asthma, neurodevelopmental and behavioral disorders including attention-deficit/hyperactivity syndrome in children, as well as infertility in males.	**Well-designed studies in humans as well as in animal models are urgently needed to distinguish the pharmacological from the toxic effects of APAP and to address how prenatal APAP medication interferes with maternal immune adaptation to pregnancy, fetal immune development, pregnancy outcome and long-term children's health.**	N/A	N/A

References:

1. Rebordosa, C. *et al.* Acetaminophen use during pregnancy: effects on risk for congenital abnormalities. *Am. J. Obstet. Gynecol.* **198**, 178.e1–7 (2008).

2. Jensen, M. S. *et al.* Maternal use of acetaminophen, ibuprofen, and acetylsalicylic acid during pregnancy and risk of cryptorchidism. *Epidemiol. Camb. Mass* **21**, 779–785 (2010).

3. Feldkamp, M. L., Meyer, R. E., Krikov, S. & Botto, L. D. Acetaminophen use in pregnancy and risk of birth defects: findings from the National Birth Defects Prevention Study. *Obstet. Gynecol.* **115**, 109–115 (2010).

4. Kristensen, D. M. *et al.* Intrauterine exposure to mild analgesics is a risk factor for development of male reproductive disorders in human and rat. *Hum. Reprod. Oxf. Engl.* **26**, 235–244 (2011).
5. Wagner-Mahler, K. *et al.* Prospective study on the prevalence and associated risk factors of cryptorchidism in 6246 newborn boys from Nice area, France. *Int. J. Androl.* **34**, e499-510 (2011).
6. Snijder, C. A. *et al.* Intrauterine exposure to mild analgesics during pregnancy and the occurrence of cryptorchidism and hypospadia in the offspring: the Generation R Study. *Hum. Reprod. Oxf. Engl.* **27**, 1191–1201 (2012).
7. Lind, J. N. *et al.* Maternal medication and herbal use and risk for hypospadias: data from the National Birth Defects Prevention Study, 1997-2007. *Pharmacoepidemiol. Drug Saf.* **22**, 783–793 (2013).
8. Fisher, B. G. *et al.* Prenatal paracetamol exposure is associated with shorter anogenital distance in male infants. *Hum. Reprod. Oxf. Engl.* **31**, 2642–2650 (2016).
9. Smarr, M. M. *et al.* Urinary paracetamol and time-to-pregnancy. *Hum. Reprod. Oxf. Engl.* **31**, 2119–2127 (2016).
10. Lind, D. V. *et al.* Maternal use of mild analgesics during pregnancy associated with reduced anogenital distance in sons: a cohort study of 1027 mother-child pairs. *Hum. Reprod. Oxf. Engl.* **32**, 223–231 (2017).
11. Smarr, M. M., Kannan, K., Chen, Z., Kim, S. & Buck Louis, G. M. Male urinary paracetamol and semen quality. *Andrology* **5**, 1082–1088 (2017).
12. Interrante, J. D. *et al.* Risk comparison for prenatal use of analgesics and selected birth defects, National Birth Defects Prevention Study 1997-2011. *Ann. Epidemiol.* **27**, 645-653.e2 (2017).
13. Ernst, A. *et al.* Acetaminophen (Paracetamol) Exposure During Pregnancy and Pubertal Development in Boys and Girls From a Nationwide Puberty Cohort. *Am. J. Epidemiol.* **188**, 34–46 (2019).
14. Reel, J. R., Lawton, A. D. & Lamb, J. C. 4th. Reproductive toxicity evaluation of acetaminophen in Swiss CD-1 mice using a continuous breeding protocol. *Fundam. Appl. Toxicol. Off. J. Soc. Toxicol.* **18**, 233–239 (1992).

15. Wiger, R. *et al.* Effects of acetaminophen and hydroxyurea on spermatogenesis and sperm chromatin structure in laboratory mice. *Reprod. Toxicol. Elmsford N* **9**, 21–33 (1995).
16. Ratnasooriya, W. D. & Jayakody, J. R. Long-term administration of large doses of paracetamol impairs the reproductive competence of male rats. *Asian J. Androl.* **2**, 247–255 (2000).
17. Yano, C. L. & Dolder, H. Rat testicular structure and ultrastructure after paracetamol treatment. *Contraception* **66**, 463–467 (2002).
18. Axelstad, M. *et al.* Mixtures of endocrine-disrupting contaminants induce adverse developmental effects in preweaning rats. *Reprod. Camb. Engl.* **147**, 489–501 (2014).
19. Holm, J. B. *et al.* Aniline Is Rapidly Converted Into Paracetamol Impairing Male Reproductive Development. *Toxicol. Sci. Off. J. Soc. Toxicol.* **148**, 288–298 (2015).
20. van den Driesche, S. *et al.* Prolonged exposure to acetaminophen reduces testosterone production by the human fetal testis in a xenograft model. *Sci. Transl. Med.* **7**, 288ra80 (2015).
21. Mandrup, K. R. *et al.* Mixtures of environmentally relevant endocrine disrupting chemicals affect mammary gland development in female and male rats. *Reprod. Toxicol. Elmsford N* **54**, 47–57 (2015).
22. Boberg, J. *et al.* Perinatal exposure to mixtures of anti-androgenic chemicals causes proliferative lesions in rat prostate. *The Prostate* **75**, 126–140 (2015).
23. Holm, J. B. *et al.* Intrauterine Exposure to Paracetamol and Aniline Impairs Female Reproductive Development by Reducing Follicle Reserves and Fertility. *Toxicol. Sci. Off. J. Soc. Toxicol.* **150**, 178–189 (2016).
24. Dean, A. *et al.* Analgesic exposure in pregnant rats affects fetal germ cell development with inter-generational reproductive consequences. *Sci. Rep.* **6**, 19789 (2016).
25. Johansson, H. K. L. *et al.* Perinatal exposure to mixtures of endocrine disrupting chemicals reduces female rat follicle reserves and accelerates reproductive aging. *Reprod. Toxicol. Elmsford N* **61**, 186–194 (2016).

26. Hay-Schmidt, A. *et al.* Prenatal exposure to paracetamol/acetaminophen and precursor aniline impairs masculinisation of male brain and behaviour. *Reprod. Camb. Engl.* **154**, 145–152 (2017).

27. Axelstad, M. *et al.* EDC IMPACT: Reduced sperm counts in rats exposed to human relevant mixtures of endocrine disrupters. *Endocr. Connect.* **7**, 139–148 (2018).

28. Rossitto, M. *et al.* In utero exposure to acetaminophen and ibuprofen leads to intergenerational accelerated reproductive aging in female mice. *Commun. Biol.* **2**, 310 (2019).

29. Rossitto, M. *et al.* Intergenerational effects on mouse sperm quality after in utero exposure to acetaminophen and ibuprofen. *FASEB J. Off. Publ. Fed. Am. Soc. Exp. Biol.* **33**, 339–357 (2019).

30. Pereira, M. R. F. *et al.* Can maternal exposure to paracetamol impair reproductive parameters of male rat offspring? *Reprod. Toxicol. Elmsford N* **93**, 68–74 (2020).

31. Kristensen, D. M. *et al.* Paracetamol (acetaminophen), aspirin (acetylsalicylic acid) and indomethacin are anti-androgenic in the rat foetal testis. *Int. J. Androl.* **35**, 377–384 (2012).

32. Albert, O. *et al.* Paracetamol, aspirin and indomethacin display endocrine disrupting properties in the adult human testis in vitro. *Hum. Reprod. Oxf. Engl.* **28**, 1890–1898 (2013).

33. Mazaud-Guittot, S. *et al.* Paracetamol, aspirin, and indomethacin induce endocrine disturbances in the human fetal testis capable of interfering with testicular descent. *J. Clin. Endocrinol. Metab.* **98**, E1757-67 (2013).

34. Gaudriault, P. *et al.* Endocrine Disruption in Human Fetal Testis Explants by Individual and Combined Exposures to Selected Pharmaceuticals, Pesticides, and Environmental Pollutants. *Environ. Health Perspect.* **125**, 087004 (2017).

35. Hurtado-Gonzalez, P. *et al.* Effects of Exposure to Acetaminophen and Ibuprofen on Fetal Germ Cell Development in Both Sexes in Rodent and Human Using Multiple Experimental Systems. *Environ. Health Perspect.* **126**, 047006 (2018).

36. Manku, G., Papadopoulos, P., Boisvert, A. & Culty, M. Cyclooxygenase 2 (COX2) Expression and prostaglandin synthesis in Neonatal Rat Testicular Germ Cells: Effects of Acetaminophen and Ibuprofen. *Andrology* (2019) doi:10.1111/andr.12727.

37. Brandlistuen, R. E., Ystrom, E., Nulman, I., Koren, G. & Nordeng, H. Prenatal paracetamol exposure and child neurodevelopment: a sibling-controlled cohort study. *Int. J. Epidemiol.* (2013) doi:10.1093/ije/dyt183.

38. Liew, Z., Ritz, B., Rebordosa, C., Lee, P. C. & Olsen, J. Acetaminophen Use During Pregnancy, Behavioral Problems, and Hyperkinetic Disorders. *JAMA Pediatr.* (2014) doi:10.1001/jamapediatrics.2013.4914; 10.1001/jamapediatrics.2013.4914.

39. Thompson, J. M. D., Waldie, K. E., Wall, C. R., Murphy, R. & Mitchell, E. A. Associations between acetaminophen use during pregnancy and ADHD symptoms measured at ages 7 and 11 years. *PloS One* **9**, e108210 (2014).

40. Liew, Z., Ritz, B., Virk, J. & Olsen, J. Maternal use of acetaminophen during pregnancy and risk of autism spectrum disorders in childhood: A Danish national birth cohort study. *Autism Res. Off. J. Int. Soc. Autism Res.* (2015) doi:10.1002/aur.1591 [doi].

41. Liew, Z., Bach, C. C., Asarnow, R. F., Ritz, B. & Olsen, J. Paracetamol use during pregnancy and attention and executive function in offspring at age 5 years. *Int. J. Epidemiol.* (2016) doi:dyw296 [pii].

42. Liew, Z., Ritz, B., Virk, J., Arah, O. A. & Olsen, J. Prenatal Use of Acetaminophen and Child IQ: a Danish Cohort Study. *Epidemiol. Camb. Mass* (2016) doi:10.1097/EDE.0000000000000540 [doi].

43. Stergiakouli, E., Thapar, A. & Davey Smith, G. Association of Acetaminophen Use During Pregnancy With Behavioral Problems in Childhood: Evidence Against Confounding. *JAMA Pediatr.* **170**, 964–970 (2016).

44. Avella-Garcia, C. B. *et al.* Acetaminophen use in pregnancy and neurodevelopment: attention function and autism spectrum symptoms. *Int. J. Epidemiol.* (2016) doi:dyw115 [pii].

45. Vlenterie, R. *et al.* Neurodevelopmental problems at 18 months among children exposed to paracetamol in utero: a propensity score matched cohort study. *Int. J. Epidemiol.* **45**, 1998–2008 (2016).

46. Bornehag, C. G. *et al.* Prenatal exposure to acetaminophen and children's language development at 30 months. *Eur. Psychiatry J. Assoc. Eur. Psychiatr.* (2017) doi:S0924-9338(17)32989-9 [pii].

47. Ystrom, E. *et al.* Prenatal Exposure to Acetaminophen and Risk of ADHD. *Pediatrics* **140**, (2017).

48. Skovlund, E., Handal, M., Selmer, R., Brandlistuen, R. E. & Skurtveit, S. Language competence and communication skills in 3-year-old children after prenatal exposure to analgesic opioids. *Pharmacoepidemiol. Drug Saf.* **26**, 625–634 (2017).

49. Gervin, K., Nordeng, H., Ystrom, E., Reichborn-Kjennerud, T. & Lyle, R. Long-term prenatal exposure to paracetamol is associated with DNA methylation differences in children diagnosed with ADHD. *Clin. Epigenetics* **9**, 77-017-0376–9. eCollection 2017 (2017).

50. Tovo-Rodrigues, L. *et al.* Is intrauterine exposure to acetaminophen associated with emotional and hyperactivity problems during childhood? Findings from the 2004 Pelotas birth cohort. *BMC Psychiatry* **18**, 368 (2018).

51. Petersen, T. G. *et al.* Use of paracetamol, ibuprofen or aspirin in pregnancy and risk of cerebral palsy in the child. *Int. J. Epidemiol.* **47**, 121–130 (2018).

52. Laue, H. E. *et al.* Association between meconium acetaminophen and childhood neurocognitive development in GESTE, a Canadian cohort study. *Toxicol. Sci. Off. J. Soc. Toxicol.* (2018) doi:10.1093/toxsci/kfy222.

53. Ruisch, I. H., Buitelaar, J. K., Glennon, J. C., Hoekstra, P. J. & Dietrich, A. Pregnancy risk factors in relation to oppositional-defiant and conduct disorder symptoms in the Avon Longitudinal Study of Parents and Children. *J. Psychiatr. Res.* **101**, 63–71 (2018).

54. Rifas-Shiman, S. L. *et al.* Associations of prenatal or infant exposure to acetaminophen or ibuprofen with mid-childhood executive function and behaviour. *Paediatr. Perinat. Epidemiol.* (2019) doi:10.1111/ppe.12596.

55. Leppert, B. *et al.* Association of Maternal Neurodevelopmental Risk Alleles With Early-Life Exposures. *JAMA Psychiatry* (2019) doi:10.1001/jamapsychiatry.2019.0774.

56. Tronnes, J. N., Wood, M., Lupattelli, A., Ystrom, E. & Nordeng, H. Prenatal paracetamol exposure and neurodevelopmental outcomes in preschool-aged children. *Paediatr. Perinat. Epidemiol.* (2019) doi:10.1111/ppe.12568.

57. Ji, Y. *et al.* Maternal Biomarkers of Acetaminophen Use and Offspring Attention Deficit Hyperactivity Disorder. *Brain Sci.* **8**, (2018).

58. Liew, Z. *et al.* Use of Negative Control Exposure Analysis to Evaluate Confounding: An Example of Acetaminophen Exposure and Attention-Deficit/Hyperactivity Disorder in Nurses' Health Study II. *Am. J. Epidemiol.* **188**, 768–775 (2019).

59. Golding, J. *et al.* Associations between paracetamol (acetaminophen) intake between 18 and 32 weeks gestation and neurocognitive outcomes in the child: A longitudinal cohort study. *Paediatr. Perinat. Epidemiol.* (2019) doi:10.1111/ppe.12582.

60. Chen, M.-H. *et al.* Prenatal Exposure to Acetaminophen and the Risk of Attention-Deficit/Hyperactivity Disorder: A Nationwide Study in Taiwan. *J. Clin. Psychiatry* **80**, (2019).

61. Bertoldi, A. D. *et al.* Associations of acetaminophen use during pregnancy and the first year of life with neurodevelopment in early childhood. *Paediatr. Perinat. Epidemiol.* (2020) doi:10.1111/ppe.12632.

62. Tovo-Rodrigues, L. *et al.* Low neurodevelopmental performance and behavioural/emotional problems at 24 and 48 months in Brazilian children exposed to acetaminophen during foetal development. *Paediatr. Perinat. Epidemiol.* **34**, 278–286 (2020).

63. Baker, B. H. *et al.* Association of Prenatal Acetaminophen Exposure Measured in Meconium With Risk of Attention-Deficit/Hyperactivity Disorder Mediated by Frontoparietal Network Brain Connectivity. *JAMA Pediatr.* (2020) doi:10.1001/jamapediatrics.2020.3080.

64. Schultz, S. T. *et al.* Acetaminophen (paracetamol) use, measles-mumps-rubella vaccination, and autistic disorder: the results of a parent survey. *Autism Int. J. Res. Pract.* **12**, 293–307 (2008).

65. Bauer, A. Z. & Kriebel, D. Prenatal and perinatal analgesic exposure and autism: an ecological link. *Environ. Health Glob. Access Sci. Source* **12**, 41 (2013).

66. Parker, S. E., Collett, B. R. & Werler, M. M. Maternal acetaminophen use during pregnancy and childhood behavioural problems: Discrepancies between mother- and teacher-reported outcomes. *Paediatr. Perinat. Epidemiol.* (2019) doi:10.1111/ppe.12601.

67. Juujarvi, S. *et al.* Follow-up study of the early, randomised paracetamol trial to preterm infants, found no adverse reactions at the two-years corrected age. *Acta Paediatr. Oslo Nor. 1992* (2018) doi:10.1111/apa.14614.

68. Jetten, M. J. A. *et al.* 'Omics analysis of low dose acetaminophen intake demonstrates novel response pathways in humans. *Toxicol. Appl. Pharmacol.* **259**, 320–328 (2012).

69. Cohen, I. V. *et al.* Acetaminophen (Paracetamol) Use Modifies the Sulfation of Sex Hormones. *EBioMedicine* (2018) doi:S2352-3964(18)30037-9 [pii].

70. Wright, C. L., Hoffman, J. H. & McCarthy, M. M. Evidence that inflammation promotes estradiol synthesis in human cerebellum during early childhood. *Transl. Psychiatry* **9**, 58 (2019).

71. Addo, K. A., Palakodety, N. & Fry, R. C. Acetaminophen modulates the expression of steroidogenesis-associated genes and estradiol levels in human placental JEG-3 cells. *Toxicol. Sci. Off. J. Soc. Toxicol.* (2020) doi:10.1093/toxsci/kfaa160.

72. Posadas, I., Santos, P., Blanco, A., Munoz-Fernandez, M. & Cena, V. Acetaminophen induces apoptosis in rat cortical neurons. *PloS One* **5**, e15360 (2010).

73. Leroux, P. *et al.* Neuroprotective effects vary across nonsteroidal antiinflammatory drugs in a mouse model of developing excitotoxic brain injury. *Neuroscience* **167**, 716–723 (2010).

74. Silva, M. H. da *et al.* Acute brain damage induced by acetaminophen in mice: effect of diphenyl diselenide on oxidative stress and mitochondrial dysfunction. *Neurotox. Res.* **21**, 334–344 (2012).

75. Gould, G. G. *et al.* Acetaminophen differentially enhances social behavior and cortical cannabinoid levels in inbred mice. *Prog. Neuropsychopharmacol. Biol. Psychiatry* **38**, 260–269 (2012).

76. Dean, S. L., Knutson, J. F., Krebs-Kraft, D. L. & McCarthy, M. M. Prostaglandin E2 is an endogenous modulator of cerebellar development and complex behavior during a sensitive postnatal period. *Eur. J. Neurosci.* **35**, 1218–1229 (2012).
77. Blecharz-Klin, K. *et al.* Paracetamol impairs the profile of amino acids in the rat brain. *Environ. Toxicol. Pharmacol.* **37**, 95–102 (2013).
78. Yisarakun, W., Supornsilpchai, W., Chantong, C., Srikiatkhachorn, A. & Maneesri-le Grand, S. Chronic paracetamol treatment increases alterations in cerebral vessels in cortical spreading depression model. *Microvasc. Res.* **94**, 36–46 (2014).
79. Viberg, H., Eriksson, P., Gordh, T. & Fredriksson, A. Paracetamol (acetaminophen) administration during neonatal brain development affects cognitive function and alters its analgesic and anxiolytic response in adult male mice. *Toxicol. Sci. Off. J. Soc. Toxicol.* **138**, 139–147 (2014).
80. Lichtensteiger, W. *et al.* Differential Gene Expression Patterns in Developing Sexually Dimorphic Rat Brain Regions Exposed to Anti-androgenic, Estrogenic, or Complex Endocrine Disruptor Mixtures: Glutamatergic Synapses as Target. *Endocrinology* en20141504 (2015) doi:10.1210/en.2014-1504 [doi].
81. Thiele, K. *et al.* Prenatal acetaminophen affects maternal immune and endocrine adaptation to pregnancy, induces placental damage, and impairs fetal development in mice. *Am. J. Pathol.* **185**, 2805–2818 (2015).
82. Saad, A. *et al.* Is There a Causal Relation between Maternal Acetaminophen Administration and ADHD? *PloS One* **11**, e0157380 (2016).
83. Zhao, W.-X. *et al.* Acetaminophen attenuates lipopolysaccharide-induced cognitive impairment through antioxidant activity. *J. Neuroinflammation* **14**, 17 (2017).
84. Blecharz-Klin, K. *et al.* Paracetamol - Effect of early exposure on neurotransmission, spatial memory and motor performance in rats. *Behav. Brain Res.* (2017) doi:S0166-4328(16)31033-6 [pii].
85. Philippot, G., Gordh, T., Fredriksson, A. & Viberg, H. Adult neurobehavioral alterations in male and female mice following developmental exposure to paracetamol (acetaminophen): characterization of a critical period. *J. Appl. Toxicol. JAT* (2017) doi:10.1002/jat.3473 [doi].
86. Blecharz-Klin, K. *et al.* Early paracetamol exposure decreases brain-derived neurotrophic factor (BDNF) in striatum and affects social behaviour and exploration in rats. *Pharmacol. Biochem. Behav.* **168**, 25–32 (2018).

87. Philippot, G. *et al.* A Cannabinoid Receptor Type 1 (CB1R) Agonist Enhances the Developmental Neurotoxicity of Acetaminophen (Paracetamol). *Toxicol. Sci. Off. J. Soc. Toxicol.* **166**, 203–212 (2018).

88. Kandis, S. *et al.* Acetaminophen (paracetamol) affects empathy-like behavior in rats: Dose-response relationship. *Pharmacol. Biochem. Behav.* **175**, 146–151 (2018).

89. Chen, Z., Wei, H., Pertovaara, A., Wang, J. & Carlson, S. Anxiety- and activity-related effects of paracetamol on healthy and neuropathic rats. *Pharmacol. Res. Perspect.* **6**, (2018).

90. Saeedan, A. S. *et al.* Effect of early natal supplementation of paracetamol on attenuation of exotoxin/endotoxin induced pyrexia and precipitation of autistic like features in albino rats. *Inflammopharmacology* **26**, 951–961 (2018).

91. Klein, R. M. *et al.* Gestational exposure to paracetamol in rats induces neurofunctional alterations in the progeny. *Neurotoxicol. Teratol.* **77**, 106838 (2019).

92. Blecharz-Klin, K. *et al.* Hypothalamus - Response to early paracetamol exposure in male rats offspring. *Int. J. Dev. Neurosci. Off. J. Int. Soc. Dev. Neurosci.* **76**, 1–5 (2019).

93. Lalert, L. *et al.* Alterations in Synaptic Plasticity and Oxidative Stress Following Long-Term Paracetamol Treatment in Rat Brain. *Neurotox. Res.* (2019) doi:10.1007/s12640-019-00090-2.

94. Vigo, M. B. *et al.* Acute acetaminophen intoxication induces direct neurotoxicity in rats manifested as astrogliosis and decreased dopaminergic markers in brain areas associated with locomotor regulation. *Biochem. Pharmacol.* **170**, 113662 (2019).

95. Motawi, T. K., Ahmed, S. A., El-Boghdady, N. A., Metwally, N. S. & Nasr, N. N. Protective effects of betanin against paracetamol and diclofenac induced neurotoxicity and endocrine disruption in rats. *Biomark. Biochem. Indic. Expo. Response Susceptibility Chem.* **24**, 645–651 (2019).

96. Koehn, L., Habgood, M., Huang, Y., Dziegielewska, K. & Saunders, N. Determinants of drug entry into the developing brain. *F1000Research* **8**, 1372 (2019).

97. Schultz, S., DeSilva, M., Gu, T. T., Qiang, M. & Whang, K. Effects of the analgesic acetaminophen (Paracetamol) and its para-aminophenol metabolite on viability of mouse-cultured cortical neurons. *Basic Clin. Pharmacol. Toxicol.* **110**, 141–144 (2012).

98. Tantarungsee, N. *et al.* Upregulation of Pro-inflammatory Cytokine Expression Following Chronic Paracetamol Treatment in Astrocyte. *Neurotox. Res.* **34**, 137–146 (2018).

99. Kim, J. H. *et al.* Environmental risk factors, protective factors, and peripheral biomarkers for ADHD: an umbrella review. *Lancet Psychiatry* **7**, 955–970 (2020).

100. Masarwa, R., Platt, R. W. & Filion, K. B. Acetaminophen use during pregnancy and the risk of attention deficit hyperactivity disorder: A causal association or bias? *Paediatr. Perinat. Epidemiol.* **34**, 309–317 (2020).

101. Talge, N. M. Prenatal acetaminophen exposure and neurodevelopment: State of the evidence. *Paediatr. Perinat. Epidemiol.* **34**, 227–229 (2020).

102. Gou, X. *et al.* Association of maternal prenatal acetaminophen use with the risk of attention deficit/hyperactivity disorder in offspring: A meta-analysis. *Aust. N. Z. J. Psychiatry* **53**, 195–206 (2019).

103. Black, E. *et al.* Medication Use and Pain Management in Pregnancy: A Critical Review. *Pain Pract. Off. J. World Inst. Pain* **19**, 875–899 (2019).

104. Bauer, A. Z., Kriebel, D., Herbert, M. R., Bornehag, C.-G. & Swan, S. H. Prenatal paracetamol exposure and child neurodevelopment: A review. *Horm. Behav.* **101**, 125–147 (2018).

105. Masarwa, R. *et al.* Prenatal Exposure to Acetaminophen and Risk for Attention Deficit Hyperactivity Disorder and Autistic Spectrum Disorder: A Systematic Review, Meta-Analysis, and Meta-Regression Analysis of Cohort Studies. *Am. J. Epidemiol.* **187**, 1817–1827 (2018).

106. Arendrup, F. S., Mazaud-Guittot, S., Jegou, B. & Kristensen, D. M. EDC IMPACT: Is exposure during pregnancy to acetaminophen/paracetamol disrupting female reproductive development? *Endocr. Connect.* **7**, 149–158 (2018).

107. McCrae, J. C., Morrison, E. E., MacIntyre, I. M., Dear, J. W. & Webb, D. J. Long-term adverse effects of paracetamol - a review. *Br. J. Clin. Pharmacol.* **84**, 2218–2230 (2018).

108. Drobnis, E. Z. & Nangia, A. K. Pain Medications and Male Reproduction. *Adv. Exp. Med. Biol.* **1034**, 39–57 (2017).

109. Kilcoyne, K. R. & Mitchell, R. T. Assessing the impact of in-utero exposures: potential effects of paracetamol on male reproductive development. *Arch. Dis. Child.* **102**, 1169–1175 (2017).

110. Toda, K. Is acetaminophen safe in pregnancy? *Scand. J. Pain* **17**, 445–446 (2017).

111. Allegaert, K. & van den Anker, J. N. Perinatal and neonatal use of paracetamol for pain relief. *Semin. Fetal. Neonatal Med.* **22**, 308–313 (2017).

112. Prenatal acetaminophen use and outcomes in children. *Am. J. Obstet. Gynecol.* **216**, B14–B15 (2017).

113. Parker, W. *et al.* The role of oxidative stress, inflammation and acetaminophen exposure from birth to early childhood in the induction of autism. *J. Int. Med. Res.* **45**, 407–438 (2017).

114. Andrade, C. Use of acetaminophen (paracetamol) during pregnancy and the risk of attention-deficit/hyperactivity disorder in the offspring. *J. Clin. Psychiatry* **77**, e312-314 (2016).

115. Andrade, C. Use of acetaminophen (paracetamol) during pregnancy and the risk of autism spectrum disorder in the offspring. *J. Clin. Psychiatry* **77**, e152-154 (2016).

116. FDA. *FDA Drug Safety Communication: FDA has reviewed possible risks of pain medicine use during pregnancy*. (2015).

117. Aminoshariae, A. & Khan, A. Acetaminophen: old drug, new issues. *J. Endod.* **41**, 588–593 (2015).

118. Hoover, R. M., Hayes, V. A. G. & Erramouspe, J. Association Between Prenatal Acetaminophen Exposure and Future Risk of Attention Deficit/Hyperactivity Disorder in Children. *Ann. Pharmacother.* **49**, 1357–1361 (2015).

119. Brune, K., Renner, B. & Tiegs, G. Acetaminophen/paracetamol: A history of errors, failures and false decisions. *Eur. J. Pain Lond. Engl.* **19**, 953–965 (2015).

120. de Fays, L. *et al.* Use of paracetamol during pregnancy and child neurological development. *Dev. Med. Child Neurol.* **57**, 718–724 (2015).

121. Tiegs, G., Karimi, K., Brune, K. & Arck, P. New problems arising from old drugs: second-generation effects of acetaminophen. *Expert Rev. Clin. Pharmacol.* **7**, 655–662 (2014).
